# PAX3 in neuroblastoma: oncogenic potential, chemosensitivity and signalling pathways

**DOI:** 10.1111/jcmm.12155

**Published:** 2013-11-04

**Authors:** Wen-Hui Fang, Qiuyu Wang, Hong-Mei Li, Mashud Ahmed, Patricia Kumar, Shant Kumar

**Affiliations:** a Institute of Inflammation and Repair Faculty of Medicine and Human Sciences, The University of ManchesterManchester, UK; b School of Biology, Chemistry and Health Science, Manchester Metropolitan UniversityManchester, UK

**Keywords:** PAX3, neuroblastoma, RNA interference, cellular functions, microarray, signalling pathway

## Abstract

Transcription factor PAX3/Pax3 contributes to diverse cell lineages during embryonic development and is important in tumourigenesis. We found that PAX3 is re-expressed in neuroblastoma and malignant neuroblastic (N-type) neuroblastoma cells had significantly higher PAX3 protein expression than their benign substrate-adherent (S-type) counterparts. Knock-down of PAX3 expression by siRNA transfection resulted in persistent cell growth inhibition in both types of neuroblastoma cell, owing to G1 cell cycle arrest and progressive apoptosis. Inhibition of PAX3 expression significantly decreased the attachment of S-type SH-EP1 cells to extra-cellular matrix proteins, fibronectin, laminin and collagen IV. Migration and invasion of both neuroblastoma cell types were markedly reduced after PAX3 down-regulation. PAX3 knock-down significantly augmented the cytotoxic effect of chemotherapeutic agents, etoposide, vincristine and cisplatin, commonly used to treat neuroblastoma. Microarray analyses revealed that particularly signalling pathways involving cell cycle, apoptosis, cell adhesion, cytoskeletal remodelling and development were altered by PAX3 down-regulation. Changes in PAX3 downstream genes identified by microarray analyses were validated in 47 genes by quantitative PCR. These novel findings lead us to propose that PAX3 might contribute to oncogenic characteristics of neuroblastoma cells by regulating a variety of crucial signalling pathways.

## Introduction

Neuroblastoma, the most common extracranial solid tumour in children, arises from the sympathoadrenal lineage of the neural crest and accounts for 15% of paediatric cancer mortality 1. PAX3/Pax3 is a developmentally expressed transcription factor with spatially and temporally restricted expression patterns in the embryo and is rapidly switched off during terminal differentiation 2,3. PAX3 is re-expressed in several tumours which arise from tissues requiring PAX3 regulation during embryonic development 5–6.

As PAX3 is essential for neural crest-derived peripheral nervous system development, its re-expression might occur in the peripheral nervous system tumour, neuroblastoma. Some studies have examined PAX3 expression in neuroblastoma with contradictory results which could be because of (a) different approaches, some of them employed inadequate reagents or techniques, used to detect its expression 7–8. The study which detected PAX3 mRNA expression used a more sensitive RT-PCR technique, whereas less sensitive riboprobes were used in another study which failed to obtain detectable PAX3 signals in neuroblastoma cell lines. Also, some PAX3 antibodies available in the past were not sufficiently sensitive to detect the endogenous protein even though *in situ* hybridization could detect the PAX3 mRNA expression in the same primary tumour tissue section; (b) PAX3 produces up to seven different isoforms designated as PAX3a-h and published studies have analysed its different isoforms 9,10; (c) this inconsistency might reflect the notoriously heterogeneous nature of neuroblastoma in which three distinct cellular phenotypes with different morphological, biochemical, differentiative and tumourigenic properties have been identified among 25 different parental neuroblastoma cell lines 12. Therefore, a better understanding of the role of PAX3 in neuroblastoma requires further studies, with regard to the diversity of neuroblastoma cell phenotypes. Understanding the signalling pathways involved in pathogenesis of neuroblastoma might lead to novel targeted therapies.

## Materials and methods

### Cell culture and siRNA transfection

Human neuroblastoma cell lines SH-SY5Y, SH-EP1, LA-1-55N and LA-1-5S were kindly provided by Dr. G. W. Makin (Paterson Institute, Manchester, UK), and cultured as previously described 13. The PAX3-positive control, JR1 (human embryonic rhabdomyosarcoma cell line) cells were grown as reported 11. All cell lines were authenticated upon receipt by comparing them to the original description. One set of small interfering RNAs (siRNAs) targeting human PAX3 mRNA (PAX3 siRNA#1-PAX3 siRNA#6) or non-targeting control siRNA were purchased from Applied Biosystems (Carlsbad, CA, USA) and transiently transfected into cells using Lipofectamine 2000 (Invitrogen, Paisley, UK) according to the manufacturer’s instructions.

### Western blotting

Proteins were separated by SDS-PAGE, transferred to nitrocellulose membrane, and probed with primary antibodies against PAX3 (Developmental Studies Hybridoma Bank), α-actin (Sigma-Aldrich, Dorset, UK) and GAPDH (Applied Biosystems) followed by appropriate horseradish peroxidase-conjugated secondary antibodies (Dako, Glostrup, Denmark). The blots were developed by chemiluminescence (Amersham, Buckinghamshire, UK), and photographed by a G:Box (Syngene, Cambridge, UK). The expression of each protein was quantified by measuring the band density using GeneTools software (Syngene).

### Cell proliferation assay

Cell proliferation was measured by MTS colorimetric assay according to the manufacturer’s instructions (Promega, Southampton, UK). At 48 hrs post-transfection, cells (1.5 × 10^4^ for SH-SY5Y and 2.0 × 10^3^ for SH-EP1) were harvested and seeded in 96-well plates and monitored for 5 days using the MTS assay. The absorbances at 490 nm were measured by a plate reader (LabSystems, Helsinki, Finland).

As different cell types have varying levels of metabolic activity which may affect the relationship between cell number and absorbance, a standard curve was generated for each cell line. Aliquots of between 0 and 3.2 × 10^5^ SH-SY5Y cells and between 0 and 2.0 × 10^5^ SH-EP1 cells were plated in 96-well plates. The media were allowed to equilibrate for 1 hr under normal growth conditions before the MTS assay. Average optical densities (ODs) were calculated and plotted against cell numbers. Cell numbers were calculated from the ODs according to the standard curves.

### Cell cycle analysis and apoptosis detection

At defined time-points, cells were harvested and fixed with 70% ethanol, and then stained with propidium iodide (PI, 50 μg/ml; Beckman Coulter, High Wycombe, UK). A total of 1.0 × 10^4^ stained cells per sample were analysed by a FACScalibur flow cytometer (Becton Dickinson, Oxford, UK) and DNA content was quantified using CellQuest Pro software (Becton Dickinson). The percentages of DNA fragmentation reflecting apoptotic cells were determined by measuring the fraction of nuclei containing a hypodiploid DNA content (sub-G1 fraction). The appearance of a sub-G1 peak is a characteristic feature of apoptosis.

### Cell migration and invasion assays

Cell migration was determined using a 24-well Boyden chamber assay (Becton Dickinson). Bottom chambers were filled with DMEM-F12 medium containing 2.5% foetal calf serum (FCS) as a chemoattractant. At 72 hrs post-transfection, cells (3.0 × 10^5^ SH-SY5Y and 5.0 × 10^4^ SH-EP1) were harvested and seeded in the top chamber in basic DMEM-F12 medium and incubated for 22 hrs. Cells were prepared as above for cell invasion (migration through membrane coated with Matrigel matrix) but were seeded on to a Matrigel invasion chamber (Becton Dickinson).

To determine the number of cells migrating (uncoated membrane) or invading (Matrigel-coated membrane) through the membrane, non-migrating or non-invading cells were removed by gently wiping the top of the membrane with a cotton swab. Cells that had migrated to the underside of the membrane were fixed with 100% methanol, stained with 0.5% crystal violet, rinsed with PBS and then eluted with 2% SDS. The absorbances at 620 nm were measured on a plate reader (LabSystems) at 620 nm.

As the different cell types might absorb the crystal violet dye differently, a standard curve was generated for each cell line. Aliquots of between 0 and 3.2 × 10^5^ cells were plated in 96-well plates and allowed to attach to the plates for 6 hrs under normal growth conditions. Cells were then fixed and stained, dye eluted and ODs measured at 620 nm. Average ODs were calculated and plotted against cell numbers. Cell numbers were calculated from the ODs according to the standard curves.

### Cell attachment analysis

At 72 hrs post-transfection, a total of 6.0 × 10^4^ SH-EP1 cells were harvested and plated in fibronectin, laminin or collagen IV coated 96-well plates (Millipore, Temecula, CA, USA) according to the manufacturer’s instructions. The plates were incubated for 2 hrs and then rinsed gently with PBS to remove any unattached cells. Wells were fixed and stained, dye eluted and the absorbances measured at 620 nm as described for cell migration assay.

### Cytotoxicity induced by chemotherapeutic drugs

Etoposide and cisplatin were dissolved in dimethyl sulphoxide (DMSO) and vincristine in methanol (all from Sigma-Aldrich), stored at −20°C, further diluted in growth medium immediately before use (the final concentrations of DMSO and methanol were <0.04). The 50% inhibitory concentration (IC_50_) and IC_80_/IC_90_ of drugs were established for SH-SY5Y and SH-EP1 cells using MTS assay (Table S1). 0.04% DMSO or methanol treatment alone showed no cytotoxicity.

At 48 hrs after siRNA transfection, fresh complete medium supplemented with IC_50_ and IC_80_/IC_90_ doses of each drug was added. The cells were cultured for a further 6, 24, 48 or 72 hrs with drugs before apoptosis assays were carried out. The apoptosis induced by each drug combined with PAX3 inhibition was analysed to determine whether they produced greater effects together than expected from simple summation of their individual effects.

### Immunofluorescence for apoptosis detection

At defined time-points, cells were harvested and stained with annexin V and PI (Invitrogen) according to the manufacturer’s instructions. Nuclei were counterstained with Hoechst 33528 (0.25 μg/ml; Sigma-Aldrich). Cells were then spotted on to a microscope slide and examined using fluorescence microscopy. The percentage of positively stained cells was determined by scoring ∼200 cells in randomly selected fields for each sample. Unstained cells were viable cells, annexin V but no PI staining represented early apoptotic cells, and annexin-V plus PI staining represented late apoptotic/necrotic cells.

### Microarray analysis

Total RNA from pools of SH-SY5Y and SH-EP1 cells transfected with PAX3 siRNA#4 and cells transfected with control siRNA were extracted at 48 hrs post-transfection using Trizol (Invitrogen). Samples were hybridized to HG-U133 PLUS2 oligonucleotide array (Affymetrix, High Wycombe, UK) according to Affymetrix protocols. Differentially expressed genes picked for subsequent analysis met the following criteria: statistical significance (probability of positive log ratio <0.1 or >0.9), a minimum normalized expression value of greater than 50, and fold change of at least of 2.0.

### Real time quantitative PCR (RT-qPCR)

Total RNA was isolated as described for microarray samples and used as a template for reverse transcription using SuperScript II™ reverse transcriptase (Invitrogen) following the manufacturer’s guidelines. Using the Roche Online Assay Design Centre, specific primers and the associated probes were selected for genes of interest (Table S2). Samples were amplified in triplicate using a LightCycler 480 Instrument (Roche, Hertfordshire, UK) as previously reported 14. Experiments were performed twice using two independent transfections.

### Statistical analysis

Data are presented as mean ± SD. A two-tailed Student’s *t*-test was used to assess statistical differences with *P < 0.05; **P < 0.01. Correlation coefficients between variables were tested by Pearson’s correlation.

## Results

### PAX3 was re-expressed in neuroblastoma cell lines

**Figure 1:**
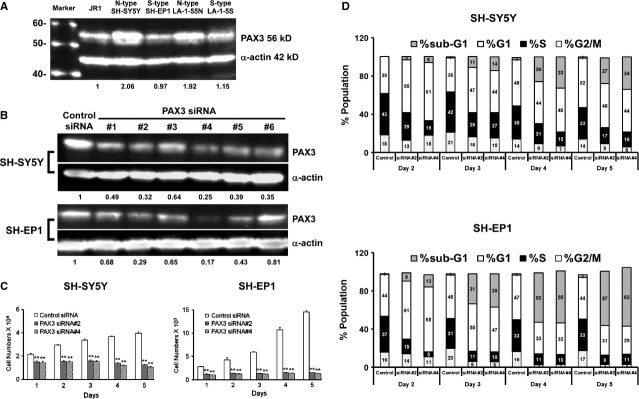
(A) Protein expression of PAX3 in four human parental neuroblastoma cell lines by western blotting. Human JR1 embryonic rhabdomyosarcoma cell line was used as positive control for PAX3 expression and α-actin as a loading control. (B) Immunoblots showing PAX3 protein levels in SH-SY5Y (top panel) and SH-EP1 (bottom panel) cells at 3 days after transfection with six different duplex siRNAs against PAX3 (PAX3 siRNA#1 - PAX3 siRNA#6) or non-targeting siRNA (control siRNA) with and α-actin as a loading control. (C) Two PAX3 siRNAs (PAX3 siRNA#2 and siRNA#4) with significant PAX3 knock-down show a marked decrease in cell proliferation compared with control siRNA in the MTS assay. Graphs show one of three independent experiments. Error bars represent mean ± SD from six wells. **P < 0.01 compared with control siRNA. (D) PAX3 silencing (PAX3 siRNA#2 and siRNA#4) arrested neuroblastoma cells in G1 phase and caused progressive cell death compared with the control (control siRNA). Inset values, relative percentages of cells in different cell cycle phases were means of two independent experiments.

In this study, we examined two malignant neuroblastic (N-type) and two benign substratum-adherent (S-type) neuroblastoma cell lines. N-type SH-SY5Y and S-type SH-EP1 cells were subclones of the MYCN-non-amplified SK-N-SH cell line, whereas N-type LA-1-55N and S-type LA-1-5S sublines were derived from the MYCN-amplified LA-N-1 cell line 13. Western blotting results revealed that PAX3 was expressed in these cell lines, more so in malignant N-type cells than in their more benign S-type counterparts (Fig. 1A).

In tumour cells, re-expression of developmentally regulated genes occurs frequently, but their role in the tumour development is not known. Using a RNA interference technique, the loss-of-function effects of PAX3 in neuroblastoma cells were investigated. As PAX3 is a direct transcriptional target of the MYCN oncogene 8, we chose the MYCN-non-amplified SK-N-SH cell lines, malignant N-type SH-SY5Y and benign S-type SH-EP1 cells to avoid the possible interference of MYCN after PAX3 knock-down. Compared to cells transfected with control siRNA, two PAX3 siRNAs (PAX3 siRNA#2 and siRNA#4) caused significant reduction in PAX3 expression in each cell line on day 3 after transfection (Fig. 1B). Furthermore, PAX3 siRNA#4 transfection alone consistently led to >60% reduction in PAX3 protein levels in both cell lines up to 7 days post-transfection (data not shown). According to the PAX3 silencing time course, all further functional studies were conducted between day 2 and day 7 after transfection.

### PAX3 knock-down inhibited cell proliferation

Two PAX3 siRNAs (PAX3 siRNA#2 and siRNA#4) causing significant inhibition of PAX3 expression were used to ascertain the effect of PAX3 silencing on cell proliferation. Compared to cells transfected with control siRNA, PAX3 knock-down completely inhibited proliferation of both neuroblastoma cell lines for 5 consecutive days (*P* < 0.01; Fig. 1C).

Cell cycle analysis was conducted to elucidate the mechanism of growth inhibition. After PAX3 knock-down, the cell cycle distribution shifted towards the left and cells accumulated in G1 and sub-G1 phases, including an initial significant G1 arrest and a subsequent sub-G1 apoptotic fraction, rising markedly from day 3 onwards in both cell lines (Fig. 1D).

Despite the initial onset of marked G1 arrest, PAX3 knock-down resulted in progressive increase in apoptosis, as shown by the appearance of a population of cells with sub-G1 DNA content. On day 2 post-transfection, the dominant effect of PAX3 knock-down was growth arrest and there were only 5.3% of SH-SY5Y cells and 11.8% of SH-EP1 cells in sub-G1 (Fig. 1D). The cell death gradually increased thereafter. Apoptosis became the dominant effect of PAX3 inhibition by day 5 post-transfection as 32% of SH-SY5Y cells and 60.5% of SH-EP1 cells accumulated in sub-G1, whereas less than 3% of control cells were in sub-G1. Taken together, PAX3 silencing induced growth inhibition by early G1 arrest and then caused irreversible block of proliferation through apoptosis in both neuroblastoma cell types, indicating that PAX3 is required for the growth and survival of neuroblastoma cells.

### PAX3 knock-down decreased the attachment of SH-EP1 cells to ECM proteins and inhibited cell migration and invasion

**Figure 2:**
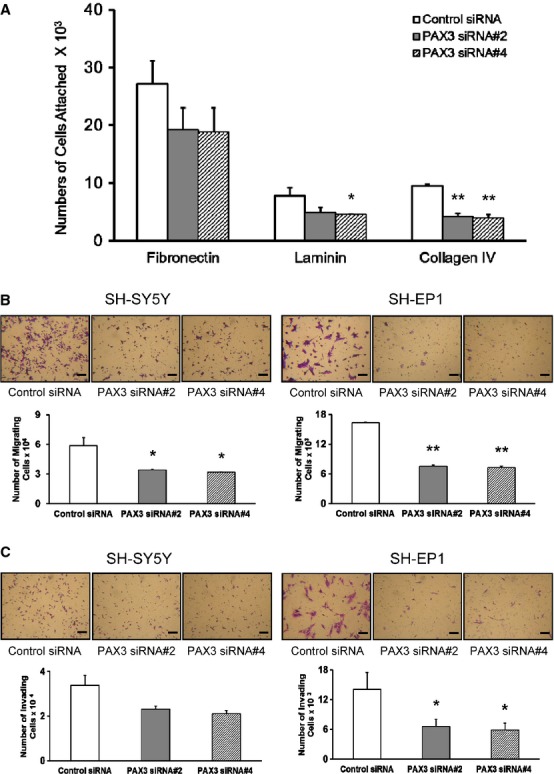
(A) Attachment of SH-EP1 cells to ECM proteins was decreased after PAX3 knock-down (PAX3 siRNA#2 and siRNA#4). (B) PAX3 silencing (PAX3 siRNA#2 and siRNA#4) inhibited migration of neuroblastoma cells. (C) PAX3 silencing (PAX3 siRNA#2 and siRNA#4) inhibited invasion of neuroblastoma cells through Matrigel. Micrograph shows a representative set of transwell filters. Scale bars represent 200 μm. The graphs are the representative example of three separate experiments. Error bars represent mean ± SD from three wells. *P < 0.05, **P < 0.01 compared with control siRNA.

Given the strong attachment of S-type cells to ECM proteins, the adhesion of SH-EP1 cells after transfection with PAX3 siRNAs was examined. PAX3 knock-down markedly decreased the attachment of SH-EP1 cells to a variety of ECM proteins, including fibronectin (30.0%), laminin (39.5%) and collagen IV (57.1%) compared with control cells (Fig. 2A). Migration of both types of neuroblastoma cell was significantly reduced after PAX3 down-regulation, 38.7% reduction in SH-SY5Y cells and 55.5% in SH-EP1 cells (Fig. 2B). PAX3 silencing potently decreased invasion in both SH-SY5Y (37.6%, *P* = 0.053) and SH-EP1 cells (58.3%, *P* = 0.037) (Fig. 2C). Thus, PAX3 signalling seems to be important for neuroblastoma metastasis.

### PAX3 knock-down potentiated the cytotoxic effect of chemotherapeutic drugs commonly used to treat neuroblastoma

**Figure 3:**
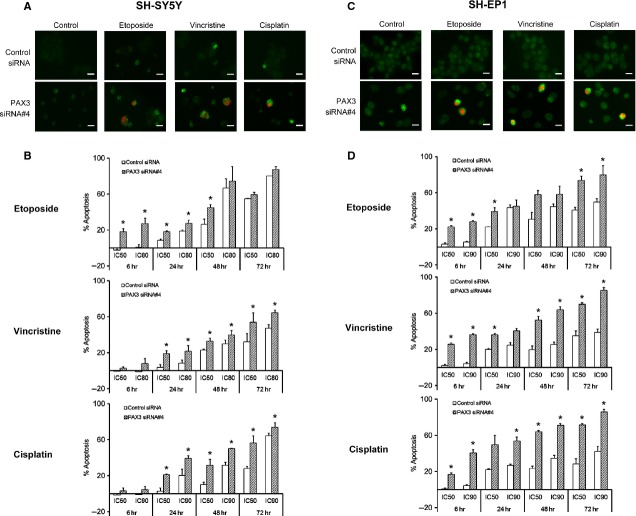
Quantification of apoptosis in PAX3 siRNA transfected SH-SY5Y (A and B) and SH-EP1 (C and D) cells following the treatment with chemotherapeutic drugs. (A) Depicts representative immunofluorescence staining of control siRNA and PAX3 siRNA#4 transfected SH-SY5Y cells which were exposed to drugs at IC_50_ for 24 hrs for annexin V (green) and propidium iodide (red). (B) Histograms show apoptosis as measured by the percentage of cells with pre-G1 DNA content among control siRNA or PAX3 siRNA#4 transfected SH-SY5Y cells treated with etoposide, vincristine and cisplatin at IC_50_ and IC_80_/IC_90_ for 6, 24, 48 and 72 hrs. (C) Depicts representative immunofluorescence staining of control siRNA and PAX3 siRNA#4 transfected SH-EP1 cells which were exposed to drugs at IC_90_ for 6 hrs for annexin V (green) and propidium iodide (red). (D) Histograms show apoptosis as measured by the percentage of cells with pre-G1 DNA content among control siRNA or PAX3 siRNA#4 transfected SH-EP1 cells treated with etoposide, vincristine and cisplatin at IC_50_ and IC_90_ for 6, 24, 48 and 72 hrs. Scale bars represent 20 μm. Error bars represent mean ± SD for two independent experiments. *P < 0.05 compared with control siRNA.

PAX3 knock-down caused an earlier and greater apoptosis induced by drugs (etoposide, vincristine and cisplatin) in both cell lines than an individual drug itself, suggesting that re-expression of PAX3 might contribute to drug resistance in neuroblastoma (Fig. 3).

Only the combination treatment with etoposide induced early apoptosis at 6 hrs of drug treatment in SH-SY5Y cells and caused a greater than additive effect (Fig. 3B). However, after prolonged treatment with etoposide, the combined treatment no longer augmented etoposide-induced cytotoxicity. With regard to vincristine and cisplatin, the combined treatment needed longer drug exposure to induce apoptosis compared with etoposide (Fig. 3B). Generally, PAX3 silencing additively complemented cytotoxicity induced by the three drugs in SH-SY5Y cells, especially at 24 hrs.

In contrast to SH-SY5Y cells, PAX3 knock-down combined with drugs caused significantly earlier apoptosis than drug treatment alone in SH-EP1 cells, with the greater than additive effect at 6 hrs of IC_90_ dosage drug exposure (*P* < 0.05–0.01; Fig. 3C and D). Therefore, benign SH-EP1 cells were more sensitive to PAX3 inhibition with the short drug exposure than their malignant counterparts. At later time-points, whereas PAX3 knock-down potentiated the sensitivity to etoposide mainly at the lower IC_50_ dosages in SH-EP1 cells, the combined treatment significantly increased apoptosis induced by vincristine and cisplatin at both IC_50_ and IC_90_ dosages (*P* < 0.05–0.01; Fig. 3D).

Our results revealed that this augmented cytotoxicity varied with the cytotoxic agent, doses, exposure time and cell phenotypes (Fig. 3). The most augmentation occurred with cisplatin, followed by vincristine and then etoposide. Furthermore, the increased apoptosis was more evident at IC_50_ dosages. PAX3 silencing combined with IC_50_ dosages of drugs even caused higher or similar levels of apoptosis than that of IC_80_/IC_90_ dosages of drugs alone. PAX3 inhibition might considerably reduce concentrations of chemotherapeutic drugs, thereby decreasing therapy-related complications such as hearing loss, cardiac dysfunction and infertility.

### PAX3 signalling pathways in neuroblastoma

Microarray results revealed that 2894 probe sets were altered, >2.00-fold, by PAX3 siRNA#4 transfection in neuroblastoma cells. After removing probe sets that belonged to the same gene, a direct comparison of the data sets for both cell lines revealed that 108 genes displayed consistent changes in both cell lines (34 up-regulated and 74 down-regulated; Table S3).

The microarray data were then compared with known PAX3 direct target genes. Some PAX3 targets, such as genes involving melanogenesis (DCT and TYRP1) and myogenesis (MYF5 and MYOD), were not expressed in the two neuroblastoma cells whereas others (RET, MET, PTEN and STX1) were expressed but not altered by PAX3 silencing 4 (Table S4). Amongst genes that changed, some were known PAX3 target genes (MITF, HES1 and NCAM1; Table 1 and Table S4), but most of them have never been reported before in the context of PAX3 signalling 5.

**Table 1:** Alterations of gene expression (fold change) in neuroblastoma cells by PAX3 knock-down assessed by microarray and qPCR

Gene symbol	Gene description	SH-EP1		SH-SY5Y	
		Microarray	q-PCR	Microarray	q-PCR
Up-regulated genes					
COL1A1	Collagen, type I, alpha 1	133.69	64.44*	1.36	6.12*
VCAN	Versican	7.05	9.73*	2.90	3.39*
IL6ST	Interleukin 6 signal transducer (gp130, oncostatin M receptor)	6.88	5.62*	2.49	3.36*
JUN	Jun oncogene	6.32	4.12*	ND	2.11*
CDKN1A	Cyclin-dependent kinase inhibitor 1A (p21Cip1)	6.31	8.05*	4.17	9.16*
DDB2	Damage-specific DNA binding protein 2	5.33	5.16*	2.35	2.40*
MDM2	Mdm2 p53 binding protein homolog	5.27	−1.09	2.33	8.99*
IGFBP3	Insulin-like growth factor binding protein 3	4.32	2.76*	1.58	1.54*
MAPK3	Mitogen-activated protein kinase 3	3.89	3.33*	1.01	2.14*
BAX	BCL2-associated X protein	2.89	2.09*	1.51	1.64*
SMAD2	SMAD family member 2	2.48	3.01*	1.81	2.07*
CDK5	Cyclin-dependent kinase 5	2.39	2.11*	−1.24	1.09
SHC1	SHC (Src homology 2 domain containing) transforming protein 1	2.33	2.99*	1.21	2.05*
NID1	Nidogen 1	2.26	1.79*	2.03	2.74*
FOXO3	Forkhead box O3	2.15	1.67*	1.85	2.52*
PDPK1	3-phosphoinositide dependent protein kinase-1	1.75	1.59*	1.82	−1.02
Down-regulated genes					
MCM7	Minichromosome maintenance complex component 7	−65.26	−7.20*	−1.74	−1.28*
GTSE1	G-2 and S-phase expressed 1	−54.15	−23.32*	−2.72	−1.41*
AURKB	Aurora kinase B	−45.82	−14.16*	−2.58	−1.45*
BIRC5	Baculoviral IAP repeat- containing 5 (survivin)	−19.69	−12.18*	−1.49	−1.42*
BUB1	BUB1 budding uninhibited by benzimidazoles 1 homologue	−38.15	−9.09*	−1.46	−1.05
AURKA	Aurora kinase A	−32.95	−7.64*	−1.46	−1.31*
CDCA3	Cell division cycle associated 3	−31.74	−12.57*	−1.76	−1.11
CENPA	Centromere protein A	−31.67	−10.39*	−1.65	−1.63*
CDC25A	Cell division cycle 25 homologue A	−22.53	−6.59*	−1.60	−1.13
HMMR	Hyaluronan-mediated motility receptor (RHAMM)	−22.39	−9.38*	−1.67	−1.35*
SKP2	S-phase kinase-associated protein 2 (p45)	−20.36	−6.33*	−1.83	−1.61*
CCNA2	Cyclin A2	−19.41	−11.99*	−1.60	−1.28*
CCNB1	Cyclin B1	−11.94	−7.67*	−1.70	−1.74*
BRCA1	Breast cancer 1	−9.88	−6.00*	−1.34	−1.27*
POLA2	Polymerase (DNA directed), alpha 2 (70kD subunit)	−8.49	−4.15*	−2.01	1.08
CALM3	Calmodulin 3	−7.69	−1.71*	−2.99	−1.03
PLK1	Polo-like kinase 1	−7.45	−12.87*	−2.80	−1.07
CDC20	Cell division cycle 20	−6.21	−9.04*	−2.03	−1.85*
CDT1	Chromatin licensing and DNA replication factor 1	−5.44	−6.85*	−2.63	1.15
TFDP1	Transcription factor Dp-1	−4.21	−2.91*	−1.70	−1.33*
H1FX	H1 histone family, member X	−3.02	−4.57*	−2.27	1.34
CDK2	Cyclin-dependent kinase 2	−2.54	−2.43*	−1.47	1.05
TUBB2C	Tubulin, beta 2C	−2.22	−3.92*	−1.68	1.00
GJA1	Gap junction protein, alpha 1	−2.11	−1.44*	−3.68	−3.54*
MITF	Microphthalmia-associated transcription factor	−1.99	−2.15*	ND	−1.20*
CAV1	Caveolin 1	−1.97	−2.15*	ND	ND
Up-regulated and Down-regulated genes					
TP53	Tumour protein p53	2.97	1.75*	−2.13	−1.71*
BCL2	B-cell CLL/lymphoma 2	1.14	−1.60	2.37	3.03
SMARCA4	SWI/SNF-related, matrix associated, actin dependent regulator of chromatin, subfamily a, member 4	−4.65	−3.61*	−1.51	2.48
GRK6	G protein-coupled receptor kinase 6	−3.81	1.47	−2.18	1.98*
CASP3	Caspase 3	−2.02	−1.20	−1.02	3.12

ND: not detected.

1.50-fold change in gene expression by PAX3 siRNA#4 transfection compared with non-targeting control siRNA was used as a threshold. Gene expression up-regulated >1.50-fold by PAX3 knock-down is shown in red; gene expression down-regulated >1.50-fold by PAX3 knock-down is shown in blue; in black means no change.

* *P* < 0.05 compared with control siRNA.

The differentially expressed genes were further mapped to a known compendium of biological and physiological pathways (GeneGo, St. Joseph, MI, USA). Using the criterion that the differentially expressed genes in that pathway are over-represented based on a hypergeometric test with *P* < 0.05, numerous signalling pathways were found to be significantly affected by PAX3 knock-down (Table S5). In particular, nine of the top 10 pathway maps are related to the cell cycle, highlighting the fact that PAX3 is closely associated with cell cycle regulation. Further analysis revealed that PAX3 mainly regulated genes involved in cell cycle, but also in apoptosis, cell adhesion, cytoskeletal remodelling, development and other signalling pathways.

To validate the microarray results, 47 genes of interest were examined by qPCR (Table 1). There was strong correlation between microarray and q-PCR data for both cells (*P* < 0.0001), confirming reliability of microarray data. The gene alterations confirmed by qPCR in neuroblastoma cells after PAX3 knock-down were then compared with three sets of microarray data from melanocytes 15, myoblasts and stem cells overexpressing PAX3 (unpublished data of our research group). The expression of 15 genes was down-regulated by PAX3 silencing but up-regulated by PAX3 overexpression, whereas five genes were up-regulated by PAX3 inhibition but down-regulated by its overexpression, indicating that these 20 genes were tightly regulated by PAX3 and therefore are strong candidates for putative PAX3 downstream genes (Table S6).

## Discussion

This is the first study that demonstrates that inhibition of PAX3 expression in neuroblastoma cells resulted in persistent cell growth inhibition, G1 cell cycle arrest, progressive apoptosis, migration and invasion inhibition and decreased attachment to ECM proteins. Moreover, PAX3 knock-down significantly sensitized neuroblastoma cells to the cytotoxic effect of chemotherapeutic drugs. Furthermore, microarray and qPCR results showed that 20 genes were tightly regulated by PAX3.

The time-dependent effects of PAX3 knock-down on cell growth demonstrated that PAX3 silencing completely inhibited proliferation of both neuroblastoma cell lines. Similarly, transfection of melanoma cell lines with PAX3 antisense oligonucleotides leads to dose-dependent inhibition of cell growth 16. Likewise, knock-down of PAX3 translocation fusion gene PAX3-forkhead (PAX3-FKHR) in alveolar rhabdomyosarcoma (ARMS) cells decreases tumour cell proliferation 17–18. Therefore, PAX3 re-expression is important for tumour cell proliferation.

Cell cycle analysis revealed that PAX3 knock-down in neuroblastoma cells initially triggered cell cycle arrest and thereafter induced progressive apoptosis. On day 2 post-transfection, 60.7% of SH-SY5Y cells and 68.1% of SH-EP1 cells accumulated in G1 phase after PAX3 silencing, compared to about 40% in control cells (Fig. 1D). Concomitantly, consistent decreases of cell percentages in S phase were observed after PAX3 knock-down, with the increase in G1:S ratio in SH-SY5Y cells (from 0.9 to 4.0) and SH-EP1 cells (from 1.2 to 13.4), indicating that PAX3 silencing inhibited the entry into S phase. This is consistent with a previous study showing that PAX3 knock-down induced G1 cell cycle arrest in B16 melanoma cells 19. Conversely, overexpression of PAX3 rescued melanoma cells from TGF-β-induced cell cycle arrest. Knock-down of PAX3-FKHR in several ARMS cell lines also caused G1 arrest, with concomitant decrease in the fraction of S phase cells, while ectopic expression of PAX3-FKHR in fibroblasts was found to accelerate the G0/G1 to S cell cycle transition 17–20. Collectively, all these findings demonstrate that PAX3 inhibition prevents the transition from G1 to S phase in different tumour cells.

**Figure 4:**
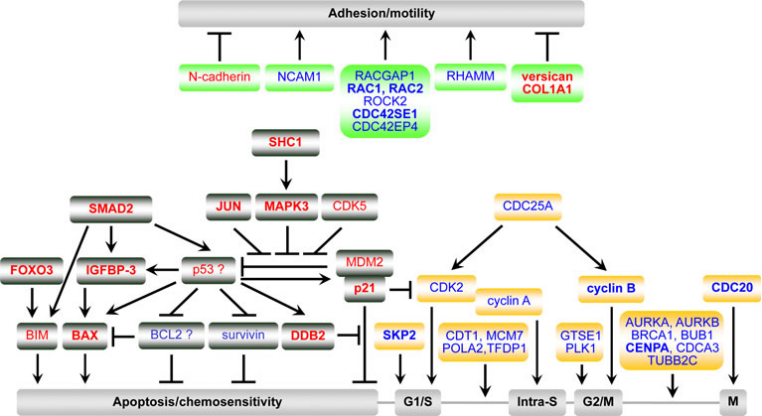
Schematic model showing how PAX3 silencing could exert its anti-cancer effects in neuroblastoma cells through regulating crucial downstream genes involved in apoptosis, cell cycle, adhesion, motility and angiogenesis. Gene expression up-regulated >1.50-fold by PAX3 siRNA#4 knock-down is shown in red; gene expression down-regulated >1.50-fold by PAX3 siRNA#4 knock-down is shown in blue; gene expression altered in both PAX3 knocked-down SH-SY5Y and SH-EP1 cells is shown in bold.

Consistent with the above results, microarray and qPCR results revealed that PAX3 knock-down has profound effects on cell cycle-related genes, as many of them were significantly altered by PAX3 silencing. In particular, 19 positive regulators and effectors of the cell cycle were down-regulated, whereas p21 and SMAD2, negative regulators, were up-regulated by PAX3 inhibition (Table 1 and Fig. 4) 21. Conversely, amongst these altered genes, 11 were up-regulated and SMAD2 was down-regulated by PAX3 overexpression in melanocytes, myoblasts and stem cells (Table S6). It is therefore possible to speculate that PAX3 silencing in neuroblastoma modulates key cell cycle regulators, thereby preventing cell cycle progression through three cell cycle checkpoints (G1/S, intra-S, and G2/M; Fig. 4).

Despite the initial onset of G1 arrest, PAX3 knock-down resulted in progressively increasing apoptosis in neuroblastoma cells. As in development, PAX3 plays an anti-apoptotic role in melanoma and RMS. Reduction in PAX3 transcripts in melanoma cell lines induces increased cell detachment and a rise in cell death from apoptosis 16,22. PAX3-FKHR also contributes to cellular survival in ARMS. Down-regulation of PAX3-FKHR by antisense oligonucleotides against PAX3 or by PAX3-KRAB transcriptional repressor in ARMS cells, caused apoptosis as assessed by sub-G1 DNA content, TUNEL or Annexin-V staining 24–25.

The PAX3-dependent survival pathway is not yet fully understood. Several targets for PAX3 have been identified, such as tumour suppressor p53, PTEN and BCL-X_L_ 5. Figure 4 shows the hierarchical apoptotic pathways induced by PAX3 silencing in neuroblastoma. PAX3 silencing activated p53 and its transcription targets, BAX, p21, MDM2 and DDB2 26–27. In addition, up-regulation of SMAD2 and IGFBP-3 by PAX3 knock-down could mediate proliferation inhibition and apoptosis *via* mediating TGF-β, p53 and BCL2 family-dependent apoptosis 28–29. Up-regulation of FOXO3 by PAX3 knock-down could also induce BIM expression and lead to apoptosis 30. Taken together, PAX3 silencing activates multiple apoptotic signalling pathways, while simultaneously inhibiting anti-apoptotic signalling pathways, thereby inducing apoptosis in neuroblastoma cells.

Notably, SH-EP1 cells appeared to be more sensitive to PAX3 down-regulation than their more malignant counterpart SH-SY5Y cells as SH-EP1 cells transfected with PAX3 siRNA demonstrated greater apoptotic effects and larger differences in differentially regulated genes compared with the latter. That might be because of the remaining amount of PAX3 in SH-SY5Y cells (0.52 in PAX3 siRNA transfected cells *versus* 2.06 in parental cells) still higher than that in SH-EP1 cells (0.17 in PAX3 siRNA transfected cells *versus* 0.97 in parental cells) after PAX3 silencing, although a reduction of over 70% was achieved in both cell lines (Fig. 1B). The effect of PAX3 down-regulation in SH-SY5Y cells appeared to be p53-independent, therefore PAX3 silencing might not exert its apoptotic effects in SH-SY5Y cells as effectively as in SH-EP1 cells. Furthermore, the expression of anti-apoptotic BCL2 was up-regulated in SH-SY5Y cells after PAX3 knock-down, which could actually counteract the apoptotic effects of PAX3 knock-down in this cell type (Table 1).

PAX3 inhibition significantly enhanced chemotherapeutic drug-induced apoptosis but the mechanism is poorly understood. We identified several potential PAX3 target genes including BCL-2, p53, survivin and FOXO3, which have been implicated in chemoresistance in neuroblastoma 31. However, the combined effects of PAX3 inhibition and chemotherapeutic drugs were tested only in MYCN-non-amplified neuroblastoma cells in this study, and MYCN is frequently amplified in advanced stage neuroblastomas with chemotherapy resistance. Further study of PAX3 inhibition in MYCN-amplified cells is therefore needed.

Up-regulation of specific genes and down-regulation of others is expected of a cell that is committed to migration. The present microarray data demonstrated that N-cadherin expression was increased and the expression of NCAM and Rho family members were down-regulated by PAX3 silencing, which could contribute to the reduced adhesion and migration through ECM proteins by PAX3 knock-down (Table S4). The expression of two ECM proteins, collagen 1A1 and versican, was up-regulated in both cells transfected with PAX3 siRNA (Table 1). Versican is anti-adhesive, belonging to the family of hyaluronan-binding proteoglycans. The mutually exclusive expression patterns of Pax3 and versican in normal embryos and versican overexpression in Pax3−/− splotch homozygotes suggest that Pax3 negatively regulates versican expression, thereby guiding neural crest cell migration 32. In addition, the hyaluronan receptor, HMMR (RHAMM), a critical regulator of cell contact and motile behaviour, was down-regulated in SH-EP1 cells after PAX3 knock-down but up-regulated in myoblasts overexpressing PAX3 (Table S6).

In summary, this study demonstrates that PAX3 contributes to the oncogenic characteristics of neuroblastoma cells. As PAX3 is predominantly expressed during embryogenesis and in malignant cells, and is absent in differentiated tissues, anti-PAX3 therapies might selectively target cancer cells, thus avoiding toxicity to normal cells. Some putative PAX3 downstream genes, such as p53, BCL2, survivin, CDC25A, PLK1 and AURKA, either have oncogenic properties or confer drug resistance and are possible targets for new anti-cancer therapeutic strategies. Further studies targeting PAX3 gene expression in MYCN-amplified neuroblastoma cells will help to define the mechanisms associated with PAX3 gene function in this tumour.

## Supporting information

**Table S1** IC50 and IC80/IC90 of chemotherapeutic drugs in neuroblastoma cells. **Table S2** Primer sequences and corresponding Roche Universal Probe Numbers for RT-qPCR analysis. **Table S3** Altered expression (fold change) of genes identified by microarray in PAX3 siRNA transfected neuroblastoma cells. **Table S4** Altered expression (fold change) of some genes of interest identified by microarray in PAX3 siRNA transfected neuroblastoma cells. **Table S5** Genes associated with biological and physiological pathways (GeneGo) significantly altered in PAX3 siRNA transfected neuroblastoma cells. **Table S6** Alterations of gene expression (fold change) in PAX3 down-regulated neuroblastoma cells compared with microarray data from PAX3 overexpressed cells.

## References

[b1] Maris JM (2010). Recent advances in neuroblastoma. N Engl J Med.

[b2] Goulding MD, Chalepakis G, Deutsch U (1991). Pax-3, a novel murine DNA binding protein expressed during early neurogenesis. EMBO J.

[b3] Lang D, Lu MM, Huang L (2005). Pax3 functions at a nodal point in melanocyte stem cell differentiation. Nature.

[b4] Relaix F, Rocancourt D, Mansouri A (2005). A Pax3/Pax7-dependent population of skeletal muscle progenitor cells. Nature.

[b5] Kubic JD, Young KP, Plummer RS (2008). Pigmentation PAX-ways: the role of Pax3 in melanogenesis, melanocyte stem cell maintenance, and disease. Pigment Cell Melanoma Res.

[b6] Wang Q, Fang WH, Krupinski J (2008). Pax genes in embryogenesis and oncogenesis. J Cell Mol Med.

[b7] Barr FG, Fitzgerald JC, Ginsberg JP (1999). Predominant expression of alternative PAX3 and PAX7 forms in myogenic and neural tumor cell line. Cancer Res.

[b8] Harris RG, White E, Phillips ES (2002). The expression of the developmentally regulated proto-oncogene Pax-3 is modulated by N-Myc. J Biol Chem.

[b9] Tsukamoto K, Nakamura Y, Niikawa N (1994). Isolation of two isoforms of the PAX3 gene transcripts and their tissue-specific alternative expression in human adult tissues. Hum Genet.

[b10] Barber TD, Barber MC, Cloutier TE (1999). PAX3 gene structure, alternative splicing and evolution. Gene.

[b11] Parker CJ, Shawcross SG, Li H (2004). Expression of PAX 3 alternatively spliced transcripts and identification of two new isoforms in human tumors of neural crest origin. Int J Cancer.

[b12] Walton JD, Kattan DR, Thomas SK (2004). Characteristics of stem cells from human neuroblastoma cell lines and in tumors. Neoplasia.

[b13] Hussein D, Holt SV, Brookes KE (2009). Preclinical efficacy of the bioreductive alkylating agent RH1 against paediatric tumours. Br J Cancer.

[b14] Carrol ED, Salway F, Pepper SD (2007). Successful downstream application of the Paxgene Blood RNA system from small blood samples in paediatric patients for quantitative PCR analysis. BMC Immunol.

[b15] Wang Q, Kumar S, Mitsios N (2007). Investigation of downstream target genes of PAX3c, PAX3e and PAX3 g isoforms in melanocytes by microarray analysis. Int J Cancer.

[b16] Muratovska A, Zhou C, He S (2003). Paired-Box genes are frequently expressed in cancer and often required for cancer cell survival. Oncogene.

[b17] Kikuchi K, Tsuchiya K, Otabe O (2008). Effects of PAX3-FKHR on malignant phenotypes in alveolar rhabdomyosarcoma. Biochem Biophys Res Commun.

[b18] Xia SJ, Holder DD, Pawel BR (2009). High expression of the PAX3-FKHR oncoprotein is required to promote tumorigenesis of human myoblasts. Am J Pathol.

[b19] Yang G, Li Y, Nishimura EK (2008). Inhibition of PAX3 by TGF-beta modulates melanocyte viability. Mol Cell.

[b20] Zhang L, Wang C (2003). PAX3-FKHR transformation increases 26 S proteasome-dependent degradation of p27Kip1, a potential role for elevated Skp2 expression. J Biol Chem.

[b21] Giono LE, Manfredi JJ (2007). Mdm2 is required for inhibition of Cdk2 activity by p21, thereby contributing to p53-dependent cell cycle arrest. Mol Cell Biol.

[b22] Scholl FA, Kamarashev J, Murmann OV (2001). PAX3 is expressed in human melanomas and contributes to tumor cell survival. Cancer Res.

[b23] He SJ, Stevens G, Braithwaite AW (2005). Transfection of melanoma cells with antisense PAX3 oligonucleotides additively complements cisplatin-induced cytotoxicity. Mol Cancer Ther.

[b24] Bernasconi M, Remppis A, Fredericks WJ (1996). Induction of apoptosis in rhabdomyosarcoma cells through down-regulation of PAX proteins. Proc Natl Acad Sci USA.

[b25] Ayyanathan K, Fredericks WJ, Berking C (2000). Hormone-dependent tumor regression *in vivo* by an inducible transcriptional repressor directed at the PAX3-FKHR oncogene. Cancer Res.

[b26] Bieging KT, Attardi LD (2012). Deconstructing p53 transcriptional networks in tumor suppression. Trends Cell Biol.

[b27] Stoyanova T, Roy N, Bhattacharjee S (2012). p21 cooperates with DDB2 protein in suppression of ultraviolet ray-induced skin malignancies. J Biol Chem.

[b28] Yu J, Zhang L, Chen A (2008). Identification of the gene transcription and apoptosis mediated by TGF-beta-Smad2/3-Smad4 signaling. J Cell Physiol.

[b29] Jogie-Brahim S, Feldman D, Oh Y (2009). Unraveling insulin-like growth factor binding protein-3 actions in human disease. Endocr Rev.

[b30] Czymai T, Viemann D, Sticht C (2010). FOXO3 modulates endothelial gene expression and function by classical and alternative mechanisms. J Biol Chem.

[b31] Goldsmith KC, Hogarty MD (2005). Targeting programmed cell death pathways with experimental therapeutics: opportunities in high-risk neuroblastoma. Cancer Lett.

[b32] Henderson DJ, Ybot-Gonzalez P, Copp AJ (1997). Over-expression of the chondroitin sulphate proteoglycan versican is associated with defective neural crest migration in the Pax3 mutant mouse (splotch). Mech Dev.

