# Supplementary material for: PAX3 in neuroblastoma: oncogenic potential, chemosensitivity and signalling pathways
Source: J Cell Mol Med. 2013 Nov 4;18(1):38–48. doi: 10.1111/jcmm.12155 (PMC3916116; doi:10.1111/jcmm.12155)
Supplement: Supplementary file 1 — Table S1 IC50 and IC80/IC90 of chemotherapeutic drugs in neuroblastoma cells. Table S2 Primer sequences and corresponding Roche Universal Probe Numbers for RT-qPCR analysis. Table S3 Altered expression (fold change) of genes identified by microarray in PAX3 siRNA transfected neuroblastoma cells. Table S4 Altered expression (fold change) of some genes of interest identified by microarray in PAX3 siRNA transfected neuroblastoma cells. Table S5 Genes associated with biological and physiological pathways (GeneGo) significantly altered in PAX3 siRNA transfected neuroblastoma cells. Table S6 Alterations of gene expression (fold change) in PAX3 down-regulated neuroblastoma cells compared with microarray data from PAX3 overexpressed cells. [file jcmm0018-0038-sd1.doc]

**Supplementary Information**

**Supplementary Table S1 IC_50_ and IC_80_/IC_90_ of chemotherapeutic drugs in neuroblastoma cells**

| **Drugs** | **SH-SY5Y** | **SH-EP1** |
| --- | --- | --- |
| Etoposide |  |  |
| IC_50_ | 2.00 ± 0.07 μmol/L | 1.56 ± 0.09 μmol/L |
| IC_80_ | 200.00 ± 4.60 μmol/L | 13.21 ± 0.91 μmol/L |
| IC_90_ | * | 35.00 ± 0.85 μmol/L |
| Vincristine |  |  |
| IC_50_ | 20.00 ± 0.47 nmol/L | 8.75 ± 0.08 nmol/L |
| IC_80_ | 80.00 ± 1.12 nmol/L | 14.29 ± 0.83 nmol/L |
| IC_90_ | * | 20.00 ± 0.35 nmol/L |
| Cisplatin |  |  |
| IC_50_ | 2.06 ± 0.15 μmol/L | 1.96 ± 0.07 μmol/L |
| IC_90_ | 13.75 ± 0.63 μmol/L | 7.35 ± 0.57 μmol/L |

*Could not be obtained.

**Supplementary Table S2** Primer sequences and corresponding Roche Universal Probe Numbers for RT-qPCR analysis

| **Gene** | **Forward primer** | **Reverse primer** | **Probe** |
| --- | --- | --- | --- |
| AURKA | gcagattttgggtggtcagt | tccgaccttcaatcatttca | 79 |
| AURKB | attgctgacttcggctggt | gtccagggtgccacacat | 69 |
| BAX | atgttttctgacggcaacttc | atcagttccggcaccttg | 104 |
| BCL2 | ttgacagaggatcatgctgtactt | atctttatttcatgaggcacgtt | 6 |
| BIRC5 | cagtgtttcttctgcttcaagg | cttattgttggtttcctttgcat | 36 |
| BRCA1 | ttgttgatgtggaggagcaa | gattccaggtaaggggttcc | 11 |
| BUB1 | ggagaacgctctgtcagca | tccaaaaactcttcagcatgag | 69 |
| CALM3 | cgatgaggaggtggatgag | tgcagtcatcatctgtacaaactc | 75 |
| CASP3 | tggaattgatgcgtgatgtt | tggctcagaagcacacaaac | 68 |
| CAV1 | acagcccagggaaacctc | gatgggaacggtgtagagatg | 42 |
| CCNA2 | ccatacctcaagtatttgccatc | tccagtctttcgtattaatgattcag | 67 |
| CCNB1 | catggtgcactttcctcctt | aggtaatgttgtagagttggtgtcc | 18 |
| CDC20 | cattcgcatctggaatgtgt | gagaccagaggatggagcac | 57 |
| CDC25A | ctccgagtcaacagattcagg | ttcaaggttttctttactgtccaa | 2 |
| CDCA3 | actgagttcccctccaaaca | gggtttctgtgggctgtct | 69 |
| CDK2 | aaagccagaaacaagttgacg | ggcacaccctcagtctcagt | 77 |
| CDK5 | gcgatgcagaaatacgagaa | ccttgaacacagttccgtagg | 60 |
| CDKN1A/p21 | tcactgtcttgtacccttgtgc | ggcgtttggagtggtagaaa | 32 |
| CDT1 | gcggagcgtctttgtgtc | agcaggtgcttctccatttc | 10 |
| CENPA | ctgcacccagtgtttctgtc | gagagtccccggtatcatcc | 15 |
| COL1A1 | gggattccctggacctaaag | ggaacacctcgctctcca | 67 |
| DDB2 | tcaaggacaaacccaccttc | aaacttcagcccagtgatgc | 78 |
| FOXO3 | gataagggcgacagcaacag | cgactatgcagtgacaggttg | 58 |
| GJA1 | gcctgaacttgccttttcat | ctccagtcacccatgttgc | 88 |
| GRK6 | gcaccgtgggttacatgg | tcagggctgaacgtgtacc | 10 |
| GTSE1 | cgctccatgagcagtgac | aagcaggaactaccaacaggag | 17 |
| H1FX | ttcggcgggatttctctt | gggggtcagagaccgagt | 19 |
| HMMR | gatgttactgctcaatttgaaagc | gccgctttttcctgtaatga | 78 |
| IGFBP3 | agaagggattttataagaaaaagcagt | tccccttggtggtgtagc | 44 |
| IL6ST | gttctgctttaataagcgagacct | attgtgccttggaggagtgt | 61 |
| JUN | ccaaaggatagtgcgatgttt | ctgtccctctccactgcaac | 62 |
| MAPK3 | ccctagcccagacagacatc | gcacagtgtccattttctaacagt | 16 |
| MCM7 | gatcctggtgtggccaag | cccggcctgttgtgtact | 17 |
| MDM2 | tccccggattagtgcgta | cggggatcattccactctc | 37 |
| MITF | agagtctgaagcaagagcactg | tgcggtcatttatgttaaatcttc | 34 |
| NID1 | cagttttcagatgagggaacg | gaaggccagtttcacagtagttg | 5 |
| PDPK1 | cgaggactgctatggcaatta | ggaggctgacagggagtg | 94 |
| PLK1 | cacagtgtcaatgcctcca | ttgctgacccagaagatgg | 30 |
| POLA2 | agacattgtttccattcaagagc | gtgtggtgtaagagttcaagagga | 35 |
| SHC1 | cggggtttcctacttggttc | ctgagtccgggtgttgaagt | 65 |
| SKP2 | ctgtctcaaggggtgattgc | ttcgataggtccatgtgctg | 44 |
| SMAD2 | gtgcaccataagaatgagttttgt | ttgttaccgtctgccttcg | 32 |
| SMARCA4 | tggaccagcactcccaag | ctggctggaactggactagag | 21 |
| TFDP1 | acgtctaacggcacaaggtt | acgtctaacggcacaaggtt | 89 |
| TP53 | aggccttggaactcaaggat | ccctttttggacttcaggtg | 12 |
| TUBB2C | ttgtctacttcctcctgcttcc | ctgatcacctcccaaaacttg | 70 |
| VCAN | gcacctgtgtgccaggata | cagggattagagtgacattcatca | 54 |
| 18S | ggagagggagcctgagaaac | tcgggagtgggtaatttgc | 8 |
| ACTB | ccaaccgcgagaagatga | ccagaggcgtacagggatag | 64 |
| GAPDH | ctgacttcaacagcgacacc | tagccaaattcgttgtcatacc | 25 |
| YWHAZ | gatccccaatgcttcacaag | tgcttgttgtgactgatcgac | 30 |

**Supplementary Table S3** Altered expression (fold change) of genes identified by microarray in PAX3 siRNA transfected neuroblastoma cells

| **Gene symbol** | **Gene name** | **SH-EP1** | **SH-SY5Y** |
| --- | --- | --- | --- |
| Up-regulated genes | | | |
| NRBP2 | nuclear receptor binding protein 2 | **28.63** | **2.15** |
| TP53INP1 | tumor protein p53 inducible nuclear protein 1 | **19.73** | **3.11** |
| GDF15 | growth differentiation factor 15 | **9.56** | **3.77** |
| ABCC5 | ATP-binding cassette, sub-family C (CFTR/MRP), member 5 | **7.62** | **2.05** |
| VCAN | versican | **7.05** | **2.90** |
| IL6ST | interleukin 6 signal transducer (gp130, oncostatin M receptor) | **6.88** | **2.49** |
| CDKN1A | cyclin-dependent kinase inhibitor 1A (p21, Cip1) | **6.31** | **4.17** |
| DDB2 | damage-specific DNA binding protein 2, 48kDa | **5.33** | **2.35** |
| MDM2 | Mdm2 p53 binding protein homolog (mouse) | **5.27** | **2.33** |
| XYLT1 | xylosyltransferase I | **5.25** | **2.51** |
| TMEM166 | transmembrane protein 166 | **4.77** | **2.26** |
| ZMAT3 | zinc finger, matrin type 3 | **4.20** | **2.35** |
| ALCAM | activated leukocyte cell adhesion molecule | **4.05** | **3.73** |
| GOPC | golgi associated PDZ and coiled-coil motif containing | **3.96** | **2.71** |
| SAR1B | SAR1 gene homolog B (S. cerevisiae) | **3.89** | **2.32** |
| MALAT1 | metastasis associated lung adenocarcinoma transcript 1 (non-protein coding) | **3.80** | **2.67** |
| LOC727770 | similar to ankyrin repeat domain 20 family, member A1 | **3.78** | **5.04** |
| SCAMP1 | secretory carrier membrane protein 1 | **3.30** | **2.31** |
| RIT1 | Ras-like without CAAX 1 | **3.17** | **2.25** |
| RRM2B | ribonucleotide reductase M2 B (TP53 inducible) | **3.10** | **2.47** |
| SESN1 | sestrin 1 | **2.81** | **2.16** |
| USP15 | ubiquitin specific peptidase 15 | **2.81** | **2.14** |
| C10orf97 | chromosome 10 open reading frame 97 | **2.81** | **2.22** |
| ASCC3 | activating signal cointegrator 1 complex subunit 3 | **2.76** | **2.84** |
| ZNF605 | zinc finger protein 605 | **2.73** | **2.25** |
| SMAD2 | SMAD family member 2 | **2.64** | **2.12** |

**(*Continued*)**

| **Gene symbol** | **Gene name** | **SH-EP1** | **SH-SY5Y** |
| --- | --- | --- | --- |
| Up-regulated genes | | | |
| SAT1 | spermidine/spermine N1-acetyltransferase 1 | **2.62** | **2.11** |
| TncRNA | trophoblast-derived noncoding RNA | **2.57** | **3.50** |
| C5orf13 | chromosome 5 open reading frame 13 | **2.50** | **2.02** |
| MBNL2 | muscleblind-like 2 (Drosophila) | **2.27** | **2.15** |
| NID1 | nidogen 1 | **2.26** | **2.03** |
| SPTLC1 | serine palmitoyltransferase, long chain base subunit 1 | **2.24** | **3.57** |
| FOXO3 | forkhead box O3 | **2.15** | **2.17** |
| C12orf5 | chromosome 12 open reading frame 5 | **2.10** | **2.19** |
| Down-regulated genes | | | |
| TACC3 | transforming, acidic coiled-coil containing protein 3 | **-68.50** | **-2.40** |
| EPR1 | effector cell peptidase receptor 1 | **-62.78** | **-8.03** |
| GTSE1 | G-2 and S-phase expressed 1 | **-54.15** | **-2.72** |
| DEPDC1B | DEP domain containing 1B | **-53.40** | **-2.24** |
| MYBL2 | v-myb myeloblastosis viral oncogene homolog (avian)-like 2 | **-53.24** | **-2.09** |
| AURKB | aurora kinase B | **-45.82** | **-2.58** |
| BIRC5 | baculoviral IAP repeat-containing 5 (survivin) | **-39.38** | **-2.77** |
| AURKA | aurora kinase A | **-32.95** | **-2.97** |
| ASF1B | ASF1 anti-silencing function 1 homolog B (S. cerevisiae) | **-29.37** | **-2.70** |
| IQGAP3 | IQ motif containing GTPase activating protein 3 | **-25.62** | **-3.23** |
| WHSC1 | Wolf-Hirschhorn syndrome candidate 1 | **-20.81** | **-3.24** |
| LONRF1 | LON peptidase N-terminal domain and ring finger 1 | **-15.07** | **-2.41** |
| RPL13A | ribosomal protein L13a | **-15.06** | **-2.07** |
| RAVER1 | ribonucleoprotein, PTB-binding 1 | **-14.30** | **-3.93** |
| CEP55 | centrosomal protein 55kDa | **-13.18** | **-2.04** |
| PCBP2 | poly(rC) binding protein 2 | **-11.40** | **-2.54** |
| SLC7A5 | solute carrier family 7 (cationic amino acid transporter, y+ system), member 5 | **-9.30** | **-2.69** |
| NEIL3 | nei endonuclease VIII-like 3 (E. coli) | **-9.13** | **-2.01** |

**(*Continued*)**

| **Gene symbol** | **Gene name** | **SH-EP1** | **SH-SY5Y** |
| --- | --- | --- | --- |
| Down-regulated genes | | | |
| POLA2 | polymerase (DNA directed), alpha 2 (70kD subunit) | **-8.49** | **-2.01** |
| CALM3 | Calmodulin 3 (phosphorylase kinase, delta) | **-7.69** | **-2.99** |
| PLK1 | polo-like kinase 1 (Drosophila) | **-7.45** | **-2.80** |
| KIAA1467 | KIAA1467 | **-7.05** | **-2.90** |
| CDC20 | cell division cycle 20 homolog (S. cerevisiae) | **-6.21** | **-2.03** |
| NUCKS1 | Nuclear casein kinase and cyclin-dependent kinase substrate 1 | **-5.85** | **-3.87** |
| CDT1 | chromatin licensing and DNA replication factor 1 | **-5.44** | **-2.63** |
| CKS2 | CDC28 protein kinase regulatory subunit 2 | **-4.82** | **-2.19** |
| HDHD1A | haloacid dehalogenase-like hydrolase domain containing 1A | **-4.72** | **-2.11** |
| HIST4H4 | histone cluster 4, H4 | **-4.52** | **-2.41** |
| NOC4L | nucleolar complex associated 4 homolog (S. cerevisiae) | **-4.32** | **-4.88** |
| DDAH1 | Dimethylarginine dimethylaminohydrolase 1 | **-4.25** | **-2.40** |
| SF3A2 | splicing factor 3a, subunit 2, 66kDa | **-4.12** | **-3.89** |
| UCP2 | uncoupling protein 2 (mitochondrial, proton carrier) | **-4.06** | **-2.33** |
| GDE1 | glycerophosphodiester phosphodiesterase 1 | **-4.02** | **-2.15** |
| TMEM109 | transmembrane protein 109 | **-3.96** | **-3.52** |
| RPL22L1 | ribosomal protein L22-like 1 | **-3.90** | **-3.08** |
| DHCR7 | 7-dehydrocholesterol reductase | **-3.83** | **-2.30** |
| GRK6 | G protein-coupled receptor kinase 6 | **-3.81** | **-2.18** |
| ND6 | NADH dehydrogenase, subunit 6 (complex I) | **-3.63** | **-2.23** |
| LOC731049 / UBE2S | ubiquitin-conjugating enzyme E2S / similar to Ubiquitin-conjugating enzyme E2S (Ubiquitin-conjugating enzyme E2-24 kDa) (Ubiquitin-protein ligase) (Ubiquitin carrier protein) (E2-EPF5) | **-3.57** | **-2.20** |
| GGA3 | golgi associated, gamma adaptin ear containing, ARF binding protein 3 | **-3.45** | **-2.62** |
| CALM1 / CALM2 / CALM3 | calmodulin 1 (phosphorylase kinase, delta) / calmodulin 2 (phosphorylase kinase, delta) / calmodulin 3 (phosphorylase kinase, delta) | **-3.42** | **-4.64** |

**(*Continued*)**

| **Gene symbol** | **Gene name** | **SH-EP1** | **SH-SY5Y** |
| --- | --- | --- | --- |
| Down-regulated genes | | | |
| EME1 | essential meiotic endonuclease 1 homolog 1 (S. pombe) | **-3.40** | **-2.46** |
| C9orf40 | chromosome 9 open reading frame 40 | **-3.31** | **-3.78** |
| H2AFX | H2A histone family, member X | **-3.30** | **-2.29** |
| RNF26 | ring finger protein 26 | **-3.26** | **-3.64** |
| LOC100130123 / NUCKS1 | nuclear casein kinase and cyclin-dependent kinase substrate 1 / PRO2870 | **-3.07** | **-2.14** |
| H1FX | H1 histone family, member X | **-3.02** | **-2.27** |
| LOC339123 | hypothetical LOC339123 | **-3.01** | **-3.85** |
| DTYMK / LOC727761 | deoxythymidylate kinase (thymidylate kinase) / similar to Deoxythymidylate kinase (thymidylate kinase) | **-2.80** | **-2.21** |
| C17orf63 | chromosome 17 open reading frame 63 | **-2.68** | **-2.02** |
| FOLR1 | folate receptor 1 (adult) | **-2.67** | **-3.21** |
| SFRS6 | splicing factor, arginine/serine-rich 6 | **-2.66** | **-2.08** |
| ANKHD1-EIF4EBP3 / EIF4EBP3 | eukaryotic translation initiation factor 4E binding protein 3 / ANKHD1-EIF4EBP3 | **-2.64** | **-2.01** |
| TMEM107 | transmembrane protein 107 | **-2.61** | **-2.12** |
| C6orf62 | chromosome 6 open reading frame 62 | **-2.59** | **-3.15** |
| KLHDC3 | kelch domain containing 3 | **-2.55** | **-2.28** |
| C12orf52 | chromosome 12 open reading frame 52 | **-2.55** | **-2.89** |
| MAZ | MYC-associated zinc finger protein (purine-binding transcription factor) | **-2.53** | **-7.83** |
| ARID1A | AT rich interactive domain 1A (SWI-like) | **-2.52** | **-2.45** |
| ANKRD40 | ankyrin repeat domain 40 | **-2.52** | **-2.19** |
| SAP30L | SAP30-like | **-2.46** | **-2.04** |
| ZNF473 | zinc finger protein 473 | **-2.43** | **-2.02** |
| HP1BP3 | Heterochromatin protein 1, binding protein 3 | **-2.28** | **-2.35** |
| SETD8 | SET domain containing (lysine methyltransferase) 8 | **-2.25** | **-4.58** |
| C20orf29 | chromosome 20 open reading frame 29 | **-2.18** | **-2.07** |
| AAAS | achalasia, adrenocortical insufficiency, alacrimia (Allgrove, triple-A) | **-2.17** | **-2.44** |
| WDR34 | WD repeat domain 34 | **-2.16** | **-2.57** |
| TIFA | TRAF-interacting protein with forkhead-associated domain | **-2.14** | **-2.34** |
| GJA1 | gap junction protein, alpha 1, 43kDa | **-2.11** | **-3.68** |

**(*Continued*)**

| **Gene symbol** | **Gene name** | **SH-EP1** | **SH-SY5Y** |
| --- | --- | --- | --- |
| Down-regulated genes | | | |
| C6orf129 | chromosome 6 open reading frame 129 | **-2.10** | **-2.41** |
| RPL37A | Ribosomal protein L37a | **-2.09** | **-7.38** |
| JUB | jub, ajuba homolog (Xenopus laevis) | **-2.08** | **-2.02** |
| RBM14 | RNA binding motif protein 14 | **-2.04** | **-2.14** |
| ST3GAL5 | ST3 beta-galactoside alpha-2,3- sialyltransferase 5 | **-2.04** | **-2.43** |

ND, not detected. 1.50-fold change of gene expression by PAX3 siRNA#4 transfection compared with non-targeting control siRNA was used as a threshold. Gene expression up-regulated > 1.50-fold by PAX3 knock-down is shown in red; gene expression down-regulated > 1.50-fold by PAX3 knock-down is shown in blue; in black means no change.

**Supplementary Table S4 Altered expression (fold change) of some genes of interest identified by microarray in PAX3 siRNA transfected neuroblastoma cells**

| **Gene symbol** | **Gene name** | **SH-EP1** | **SH-SY5Y** |
| --- | --- | --- | --- |
| Up-regulated genes | | | |
| CDH2 | cadherin 2, type 1, N-cadherin (neuronal) | **2.10** | **1.42** |
| BCL2L11/BIM | BCL2-like 11 (apoptosis facilitator) | **1.26** | **1.72** |
| HES1 | hairy and enhancer of split 1 | **ND** | **1.55** |
| Down-regulated genes | | | |
| RACGAP1 | Rac GTPase activating protein 1 | **-6.65** | **-1.35** |
| RAC2 | ras-related C3 botulinum toxin substrate 2 (rho family, small GTP binding protein Rac2) | **-2.24** | **-1.67** |
| ROCK2 | Rho-associated, coiled-coil containing protein kinase 2 | **-1.94** | **-1.05** |
| RAC1 | ras-related C3 botulinum toxin substrate 1 (rho family, small GTP binding protein Rac1) | **-1.70** | **-1.51** |
| CDC42SE1 | CDC42 small effector 1 | **-1.65** | **-2.95** |
| CDC42EP4 | CDC42 effector protein 4 | **-1.45** | **-2.29** |
| NCAM1 | neural cell adhesion molecule 1 | **ND** | **-1.53** |
| Non-altered genes | | | |
| RET | ret proto-oncogene | ND | **1.08** |
| MET | met proto-oncogene (hepatocyte growth factor receptor) | **1.16** | ND |
| PTEN | phosphatase and tensin homolog (mutated in multiple advanced cancers 1) | **1.00** | **1.18** |
| STX1A | syntaxin 1A (brain) | **ND** | **-1.20** |
| Non-expressed genes | | | |
| DCT | dopachrome tautomerase (dopachrome delta-isomerase, tyrosine-related protein 2) | **ND** | **ND** |
| TYRP1 | tyrosinase-related protein 1 | **ND** | **ND** |
| MYF5 | myogenic factor 5 | **ND** | **ND** |
| MYOD1 | myogenic differentiation 1 | **ND** | **ND** |

ND, not detected. 1.50-fold change of gene expression by PAX3 siRNA#4 transfection compared with non-targeting control siRNA was used as a threshold. Gene expression up-regulated > 1.50-fold by PAX3 knock-down is shown in red; gene expression down-regulated > 1.50-fold by PAX3 knock-down is shown in blue; in black means no change.

**Supplementary Table S5 Genes associated with biological and physiological pathways (GeneGo) significantly altered in PAX3 siRNA transfected neuroblastoma cells**

| **Map** | **Map folders** | **Cell process** | ***P*** | **Changed genes** | **Total genes** |
| --- | --- | --- | --- | --- | --- |
| Cell cycle_Start of DNA replication in early S phase | Regulatory processes/Cell cycle | [cell cycle](http://portal.genego.com/cgi/process.cgi?id=-1352108539) | 1.57E-15 | **27** | **31** |
| [Cell cycle_Role of APC in cell cycle regulation](http://portal.genego.com/cgi/imagemap.cgi?id=472&filter=1) | Regulatory processes/Cell cycle | [cell cycle](http://portal.genego.com/cgi/process.cgi?id=-1352108539) | 8.17E-15 | **27** | **32** |
| [Cell cycle_The metaphase checkpoint](http://portal.genego.com/cgi/imagemap.cgi?id=711&filter=1) | Regulatory processes/Cell cycle | [cell cycle](http://portal.genego.com/cgi/process.cgi?id=-1352108539) | 1.28E-13 | **28** | **36** |
| [Cell cycle_Chromosome condensation in prometaphase](http://portal.genego.com/cgi/imagemap.cgi?id=709&filter=1) | Regulatory processes/Cell cycle | [cell cycle](http://portal.genego.com/cgi/process.cgi?id=-1352108539) | 8.34E-13 | **19** | **20** |
| [Cell cycle_Spindle assembly and chromosome separation](http://portal.genego.com/cgi/imagemap.cgi?id=712&filter=1) | Regulatory processes/Cell cycle | [cell cycle](http://portal.genego.com/cgi/process.cgi?id=-1352108539) | 3.02E-11 | **24** | **32** |
| [Cell cycle_Role of Nek in cell cycle regulation](http://portal.genego.com/cgi/imagemap.cgi?id=731&filter=1) | Protein function/Kinases Regulatory processes/Cell cycle | [cell cycle, protein kinase cascade](http://portal.genego.com/cgi/process.cgi?id=-1352108539) | 5.71E-06 | **17** | **29** |
| [Cell cycle_Regulation of G1/S transition (part 1)](http://portal.genego.com/cgi/imagemap.cgi?id=544&filter=1) | Regulatory processes/Cell cycle | [cell cycle](http://portal.genego.com/cgi/process.cgi?id=-1352108539) | 8.12E-06 | **20** | **38** |
| [Apoptosis and survival_Role of CDK5 in neuronal death and survival](http://portal.genego.com/cgi/imagemap.cgi?id=2374&filter=1) | Regulatory processes/Apoptosis and survival Regulatory processes/ Development/Neurogenesis | [apoptosis, response to extracellular stimulus](http://portal.genego.com/cgi/process.cgi?id=-1971582702) | 1.94E-05 | **17** | **31** |
| [Development_Regulation of CDK5 in CNS](http://portal.genego.com/cgi/imagemap.cgi?id=2226&filter=1) | Protein function/G-proteins/GPCR Protein function/Growth factors Regulatory processes/Development/ Neurogenesis | [intracellular receptor-mediated signaling pathway, G-protein coupled receptor protein signaling pathway, response to extracellular stimulus](http://portal.genego.com/cgi/process.cgi?id=-391082717) | 2.18E-05 | **14** | **23** |
| [Transport_RAN regulation pathway](http://portal.genego.com/cgi/imagemap.cgi?id=404&filter=1) | Protein function/G-proteins/RAS-group Regulatory processes/Transport | [small GTPase mediated signal transduction](http://portal.genego.com/cgi/process.cgi?id=-1779154733) | 2.33E-05 | **12** | **18** |
| [Cell cycle_Cell cycle (generic schema)](http://portal.genego.com/cgi/imagemap.cgi?id=645&filter=1) | Regulatory processes/Cell cycle | [cell cycle](http://portal.genego.com/cgi/process.cgi?id=-1352108539) | 3.37E-05 | **13** | **21** |

(*Continued*)

| **Map** | **Map folders** | **Cell process** | ***P*** | **Changed genes** | **Total genes** |
| --- | --- | --- | --- | --- | --- |
| [Development_Beta-adrenergic receptors regulation of ERK](http://portal.genego.com/cgi/imagemap.cgi?id=2436&filter=1) | Disease maps/Cardiac Hypertrophy Protein function/G-proteins/GPCR Regulatory processes/Development (common pathways) | [G-protein coupled receptor protein signaling pathway, response to extracellular stimulus](http://portal.genego.com/cgi/process.cgi?id=-513419078) | 5.65E-05 | **17** | **33** |
| [Development_FGFR signaling pathway](http://portal.genego.com/cgi/imagemap.cgi?id=444&filter=1) | Protein function/Growth factors Regulatory processes/Development (common pathways) | [intracellular receptor-mediated signaling pathway, response to extracellular stimulus](http://portal.genego.com/cgi/process.cgi?id=-391082717) | 8.28E-05 | **20** | **43** |
| [Transcription_Ligand-dependent activation of the ESR1/SP pathway](http://portal.genego.com/cgi/imagemap.cgi?id=2208&filter=1) | Protein function/Hormones Protein function/Transcription factors | [response to hormone stimulus, transcription](http://portal.genego.com/cgi/process.cgi?id=-719123304) | 1.47E-04 | **15** | **29** |
| [DNA damage_ATM/ATR regulation of G1/S checkpoint](http://portal.genego.com/cgi/imagemap.cgi?id=426&filter=1) | Regulatory processes/Cell cycle Regulatory processes/DNA-damage | [cell cycle](http://portal.genego.com/cgi/process.cgi?id=-1352108539) | 1.49E-04 | **16** | **32** |
| [Membrane-bound ESR1: interaction with G-proteins signaling](http://portal.genego.com/cgi/imagemap.cgi?id=2212&filter=1) | Protein function/G-proteins Protein function/Hormones Regulatory processes/Development (common pathways) | [response to hormone stimulus, response to extracellular stimulus](http://portal.genego.com/cgi/process.cgi?id=-719123304) | 2.25E-04 | **17** | **36** |
| [DNA damage_Brca1 as a transcription regulator](http://portal.genego.com/cgi/imagemap.cgi?id=525&filter=1) | Protein function/Transcription factors Regulatory processes/DNA-damage | [transcription](http://portal.genego.com/cgi/process.cgi?id=-929449585) | 2.40E-04 | **15** | **30** |
| [dATP/dITP metabolism](http://portal.genego.com/cgi/imagemap.cgi?id=865&filter=1) | Metabolic maps (common pathways)/Nucleotide metabolism |  | 2.91E-04 | **22** | **53** |
| [Immune response_Oncostatin M signaling via MAPK in human cells](http://portal.genego.com/cgi/imagemap.cgi?id=2204&filter=1) | Protein function/Cyto/chemokines Regulatory processes/Immune response: organism-specific maps for Mouse, Rat and Human | [cytokine and chemokine mediated signaling pathway, immune response](http://portal.genego.com/cgi/process.cgi?id=-2012554208) | 3.40E-04 | **17** | **37** |

(*Continued*)

| **Map** | **Map folders** | **Cell process** | ***P*** | **Changed genes** | **Total genes** |
| --- | --- | --- | --- | --- | --- |
| [TTP metabolism](http://portal.genego.com/cgi/imagemap.cgi?id=872&filter=1) | Metabolic maps (common pathways)/Nucleotide metabolism |  | 5.01E-04 | **17** | **38** |
| [Immune response _Oncostatin M signaling via MAPK in mouse cells](http://portal.genego.com/cgi/imagemap.cgi?id=2205&filter=1) | Protein function/Cyto/chemokines Regulatory processes/Immune response/Immune response: organism-specific maps for Mouse, Rat and Human | [cytokine and chemokine mediated signaling pathway, immune response](http://portal.genego.com/cgi/process.cgi?id=-2012554208) | 5.44E-04 | **16** | **35** |
| [DNA damage_ATM / ATR regulation of G2 / M checkpoint](http://portal.genego.com/cgi/imagemap.cgi?id=441&filter=1) | Regulatory processes/Cell cycle Regulatory processes/DNA-damage | [cell cycle](http://portal.genego.com/cgi/process.cgi?id=-1352108539) | 6.28E-04 | **13** | **26** |
| [Cell cycle_Regulation of G1/S transition (part 2)](http://portal.genego.com/cgi/imagemap.cgi?id=474&filter=1) | Regulatory processes/Cell cycle | [cell cycle](http://portal.genego.com/cgi/process.cgi?id=-1352108539) | 6.28E-04 | **13** | **26** |
| [Immune response_PGE2 common pathways](http://portal.genego.com/cgi/imagemap.cgi?id=2386&filter=1) | Regulatory processes/Immune response | [immune response](http://portal.genego.com/cgi/process.cgi?id=-195107408) | 9.38E-04 | **14** | **30** |
| [Development_Mu-type opioid receptor signaling via Beta-arrestin](http://portal.genego.com/cgi/imagemap.cgi?id=2452&filter=1) | Protein function/G-proteins/GPCR Regulatory processes/Development/ Neurogenes | [G-protein coupled receptor protein signaling pathway, response to extracellular stimulus](http://portal.genego.com/cgi/process.cgi?id=-513419078) | 1.01E-03 | **11** | **21** |
| [Development_Alpha-2 adrenergic receptor activation of ERK](http://portal.genego.com/cgi/imagemap.cgi?id=2427&filter=1) | Protein function/G-proteins/GPCR Regulation of metabolism/Regulation of lipid metabolism Regulatory processes/Development (common pathways) | [G-protein coupled receptor protein signaling pathway, response to extracellular stimulus](http://portal.genego.com/cgi/process.cgi?id=-513419078) | 1.03E-03 | **17** | **40** |
| Development_PDGF signaling via MAPK cascades | Protein function/Growth factors Regulatory processes/Development (common pathways) | [intracellular receptor- mediated signaling pathway, response to extracellular stimulus](http://portal.genego.com/cgi/process.cgi?id=-391082717) | 1.28E-03 | **15** | **34** |

**(*Continued*)**

| **Map** | **Map folders** | **Cell process** | ***P*** | **Changed genes** | **Total genes** |
| --- | --- | --- | --- | --- | --- |
| [Cytoskeleton remodeling_TGF, WNT and cytoskeletal remodeling](http://portal.genego.com/cgi/imagemap.cgi?id=715&filter=1) | Regulatory processes/Cytoskeleton remodeling |  | 1.35E-03 | **35** | **107** |
| [Cell cycle_Initiation of mitosis](http://portal.genego.com/cgi/imagemap.cgi?id=442&filter=1) | Regulatory processes/Cell cycle | [cell cycle](http://portal.genego.com/cgi/process.cgi?id=-1352108539) | 1.60E-03 | **12** | **25** |
| [Apoptosis and survival_HTR1A signaling](http://portal.genego.com/cgi/imagemap.cgi?id=2947&filter=1) | Protein function/G-proteins/GPCR Regulatory processes/Apoptosis and survival | [apoptosis, G-protein coupled receptor protein signaling pathway](http://portal.genego.com/cgi/process.cgi?id=-1971582702) | 1.62E-03 | **16** | **38** |
| [dGTP metabolism](http://portal.genego.com/cgi/imagemap.cgi?id=890&filter=1) | Metabolic maps/Metabolic maps (common pathways)/Nucleotide metabolism |  | 1.62E-03 | **16** | **38** |
| [wtCFTR and delta508 traffic / Clathrin coated vesicles formation (norm and CF)](http://portal.genego.com/cgi/imagemap.cgi?id=2672&filter=1) | Disease maps/Cystic fibrosis |  | 1.64E-03 | **10** | **19** |
| [Development_Delta- and kappa-type opioid receptors signaling via beta-arrestin](http://portal.genego.com/cgi/imagemap.cgi?id=2735&filter=1) | Protein function/G-proteins/GPCR Regulatory processes/Development/ Neurogenesis | [G-protein coupled receptor protein signaling pathway, response to extracellular stimulus](http://portal.genego.com/cgi/process.cgi?id=-513419078) | 1.64E-03 | **10** | **19** |
| [Development_Beta-adrenergic receptors signaling via beta-arrestin](http://portal.genego.com/cgi/imagemap.cgi?id=2444&filter=1) | Protein function/G-proteins/GPCR Regulatory processes/Development/ Development (common pathways) | [G-protein coupled receptor protein signaling pathway, response to extracellular stimulus](http://portal.genego.com/cgi/process.cgi?id=-513419078) | 1.66E-03 | **11** | **22** |
| [ATP/ITP metabolism](http://portal.genego.com/cgi/imagemap.cgi?id=873&filter=1) | Metabolic maps/Metabolic maps (common pathways)/Nucleotide metabolism |  | 1.77E-03 | **26** | **74** |
| [Transcription_CREB pathway](http://portal.genego.com/cgi/imagemap.cgi?id=409&filter=1) | Protein function/Transcription factors | [transcription](http://portal.genego.com/cgi/process.cgi?id=-929449585) | 1.82E-03 | **15** | **35** |

**(*Continued*)**

| **Map** | **Map folders** | **Cell process** | ***P*** | **Changed genes** | **Total genes** |
| --- | --- | --- | --- | --- | --- |
| [Development_TPO in cell process](http://portal.genego.com/cgi/imagemap.cgi?id=631&filter=1) | Protein function/Growth factors Regulatory processes/ Development/Hemopoiesis | [intracellular receptor-mediated signaling pathway, response to extracellular stimulus](http://portal.genego.com/cgi/process.cgi?id=-391082717) | 1.97E-03 | **17** | **42** |
| [Signal transduction_PTEN pathway](http://portal.genego.com/cgi/imagemap.cgi?id=676&filter=1) | Protein function/Phosphatases | [protein amino acid dephosphorylation](http://portal.genego.com/cgi/process.cgi?id=-2102910324) | 1.97E-03 | **17** | **42** |
| [Development_Beta-adrenergic receptors transactivation of EGFR](http://portal.genego.com/cgi/imagemap.cgi?id=2433&filter=1) | Protein function/G-proteins/GPCR Regulatory processes/ Development/Epidermal cell development | [G-protein coupled receptor protein signaling pathway, response to extracellular stimulus](http://portal.genego.com/cgi/process.cgi?id=-513419078) | 2.03E-03 | **14** | **32** |
| [Development_Alpha-1 adrenergic receptors signaling via cAMP](http://portal.genego.com/cgi/imagemap.cgi?id=2394&filter=1) | Protein function/G-proteins/GPCR Regulatory processes/Development (common pathways) | [G-protein coupled receptor protein signaling pathway, response to extracellular stimulus](http://portal.genego.com/cgi/process.cgi?id=-513419078) | 2.49E-03 | **8** | **14** |
| [Development_G-Proteins mediated regulation MARK-ERK signaling](http://portal.genego.com/cgi/imagemap.cgi?id=463&filter=1) | Protein function/G-proteins/GPCR Regulatory processes/Development/Development (common pathways) | [G-protein coupled receptor protein signaling pathway, response to extracellular stimulus](http://portal.genego.com/cgi/process.cgi?id=-513419078) | 2.56E-03 | **15** | **36** |
| [Development_Dopamine D2 receptor transactivation of EGFR](http://portal.genego.com/cgi/imagemap.cgi?id=2457&filter=1) | Protein function/G-proteins/GPCR Regulatory processes/Development/Neurogenes /Oxidative stress | [G-protein coupled receptor protein signaling pathway, response to extracellular stimulus](http://portal.genego.com/cgi/process.cgi?id=-513419078) | 2.70E-03 | **10** | **20** |
| [Development _ACM2 and ACM4 activation of ERK](http://portal.genego.com/cgi/imagemap.cgi?id=2516&filter=1) | Protein function/G-proteins/GPCR Regulatory processes/Development (common pathways) | [G-protein coupled receptor protein signaling pathway, response to extracellular stimulus](http://portal.genego.com/cgi/process.cgi?id=-513419078) | 2.89E-03 | **14** | **33** |

**(*Continued*)**

| **Map** | **Map folders** | **Cell process** | ***P*** | **Changed genes** | **Total genes** |
| --- | --- | --- | --- | --- | --- |
| [Immune response_IL2 activation and signaling pathway](http://portal.genego.com/cgi/imagemap.cgi?id=430&filter=1) | Protein function/Cyto/chemokines Regulatory processes/Immune response | [cytokine and chemokine mediated signaling pathway, immune response](http://portal.genego.com/cgi/process.cgi?id=-2012554208) | **4.02E-03** | **14** | **34** |
| [Development_EDNRB signaling](http://portal.genego.com/cgi/imagemap.cgi?id=2273&filter=1) | Disease maps/Cardiac Hypertrophy Protein function/G-proteins/GPCR Protein function/Hormones Regulatory processes/Development/ Development (common pathways) | [response to hormone stimulus, G-protein coupled receptor protein signaling pathway, response to extracellular stimulus](http://portal.genego.com/cgi/process.cgi?id=-719123304) | **4.02E-03** | **14** | **34** |
| [Transcription_Role of heterochromatin protein 1 (HP1) family in transcriptional silencing](http://portal.genego.com/cgi/imagemap.cgi?id=671&filter=1) | Regulatory processes/Transcription | [transcription](http://portal.genego.com/cgi/process.cgi?id=-929449585) | **4.24E-03** | **10** | **21** |
| [Development_Ligand-independent activation of ESR1 and ESR2](http://portal.genego.com/cgi/imagemap.cgi?id=2210&filter=1) | Protein function/Growth factors Protein function/Hormones Protein function/Transcription factors Regulatory processes/Development/ Development (common pathways) | [response to hormone stimulus, transcription, intracellular receptor-mediated signaling pathway, response to extracellular stimulus](http://portal.genego.com/cgi/process.cgi?id=-719123304) | **4.75E-03** | **15** | **38** |
| [Development_VEGF-family signaling](http://portal.genego.com/cgi/imagemap.cgi?id=445&filter=1) | Protein function/Growth factors Regulatory processes/Development/ Angiogenesis | [intracellular receptor-mediated signaling pathway, response to extracellular stimulus](http://portal.genego.com/cgi/process.cgi?id=-391082717) | **6.31E-03** | **13** | **32** |
| [Apoptosis and survival_Apoptotic Activin A signaling](http://portal.genego.com/cgi/imagemap.cgi?id=2476&filter=1) | Protein function/Growth factors Regulatory processes/Apoptosis and survival | [intracellular receptor-mediated signaling pathway, apoptosis](http://portal.genego.com/cgi/process.cgi?id=-391082717) | **6.39E-03** | **10** | **22** |

**(*Continued*)**

| **Map** | **Map folders** | **Cell process** | ***P*** | **Changed genes** | **Total genes** |
| --- | --- | --- | --- | --- | --- |
| [Development_EDG1 signaling via beta-arrestin](http://portal.genego.com/cgi/imagemap.cgi?id=2808&filter=1) | Protein function/G-proteins/GPCR Regulatory processes/Development (common pathways) | [G-protein coupled receptor protein signaling pathway, response to extracellular stimulus](http://portal.genego.com/cgi/process.cgi?id=-513419078) | **7.24E-03** | **12** | **29** |
| [Apoptosis and survival_p53-dependent apoptosis](http://portal.genego.com/cgi/imagemap.cgi?id=428&filter=1) | Protein function/Transcription factors Regulatory processes/Apoptosis and survival | [apoptosis, transcription](http://portal.genego.com/cgi/process.cgi?id=-1971582702) | **7.24E-03** | **12** | **29** |
| [Development_EGFR signaling via small GTPases](http://portal.genego.com/cgi/imagemap.cgi?id=704&filter=1) | Protein function/G-proteins/ RAS-group Protein function/Growth factors Regulatory processes/ Development/Epidermal cell development | [intracellular receptor-mediated signaling pathway, small GTPase mediated signal transduction, response to extracellular stimulus](http://portal.genego.com/cgi/process.cgi?id=-391082717) | **7.24E-03** | **12** | **29** |
| [Transcription_ChREBP regulation pathway](http://portal.genego.com/cgi/imagemap.cgi?id=464&filter=1) | Protein function/G-proteins/GPCR Protein function/Transcription factors | [G-protein coupled receptor protein signaling pathway, transcription](http://portal.genego.com/cgi/process.cgi?id=-513419078) | **7.26E-03** | **7** | **13** |
| [Normal wtCFTR traffic / ER-to-Golgi](http://portal.genego.com/cgi/imagemap.cgi?id=2669&filter=1) | Disease maps/Cystic fibrosis |  | **7.26E-03** | **7** | **13** |
| [Delta508-CFTR traffic / ER-to-Golgi in CF](http://portal.genego.com/cgi/imagemap.cgi?id=2670&filter=1) | Disease maps/Cystic fibrosis |  | **7.26E-03** | **7** | **13** |
| [Cell adhesion_Chemokines and adhesion](http://portal.genego.com/cgi/imagemap.cgi?id=716&filter=1) | Protein function/Cyto/chemokines Regulatory processes/Cell adhesion | [cell adhesion, cytokine and chemokine mediated signaling pathway](http://portal.genego.com/cgi/process.cgi?id=-552545827) | **7.31E-03** | **29** | **93** |
| [Cytoskeleton remodeling_Cytoskeleton remodeling](http://portal.genego.com/cgi/imagemap.cgi?id=714&filter=1) | Regulatory processes/Cytoskeleton remodeling |  | **7.32E-03** | **30** | **97** |

**(*Continued*)**

| **Map** | **Map folders** | **Cell process** | ***P*** | **Changed genes** | **Total genes** |
| --- | --- | --- | --- | --- | --- |
| [Development_A3 receptor signaling](http://portal.genego.com/cgi/imagemap.cgi?id=644&filter=1) | Protein function/G-proteins/GPCR Regulatory processes/Development (common pathways) | [G-protein coupled receptor protein signaling pathway, response to extracellular stimulus](http://portal.genego.com/cgi/process.cgi?id=-513419078) | **7.35E-03** | **14** | **36** |
| [Development_Angiotensin activation of ERK](http://portal.genego.com/cgi/imagemap.cgi?id=437&filter=1) | Disease maps/Cardiac Hypertrophy Protein function/G-proteins/GPCR Regulatory processes/Development/ Angiogenesis Regulatory processes/ Epidermal cell development | [response to extracellular stimulus, G-protein coupled receptor protein signaling pathway, response to extracellular stimulus](http://portal.genego.com/cgi/process.cgi?id=-1078420898) | **8.24E-03** | **11** | **26** |
| [Transcription_Role of Akt in hypoxia induced HIF1 activation](http://portal.genego.com/cgi/imagemap.cgi?id=448&filter=1) | Protein function/Kinases Protein function/Transcription factors Regulatory processes/Hypoxia response | [transcription, protein kinase cascade](http://portal.genego.com/cgi/process.cgi?id=-929449585) | **8.24E-03** | **11** | **26** |
| [Immune response _IL3 activation and signaling pathway](http://portal.genego.com/cgi/imagemap.cgi?id=657&filter=1) | Protein function/Cyto/chemokines Regulatory processes/Immune response | [cytokine and chemokine mediated signaling pathway, immune response](http://portal.genego.com/cgi/process.cgi?id=-2012554208) | **8.24E-03** | **11** | **26** |
| [Development_TGF-beta receptor signaling](http://portal.genego.com/cgi/imagemap.cgi?id=475&filter=1) | Protein function/Growth factors Regulatory processes/Development (common pathways) | [intracellular receptor-mediated signaling pathway, response to extracellular stimulus](http://portal.genego.com/cgi/process.cgi?id=-391082717) | **8.26E-03** | **15** | **40** |
| [Regulation of CFTR activity (norm and CF)](http://portal.genego.com/cgi/imagemap.cgi?id=2269&filter=1) | Disease maps/Cystic fibrosis |  | **8.26E-03** | **15** | **40** |

**(*Continued*)**

| **Map** | **Map folders** | **Cell process** | ***P*** | **Changed genes** | **Total genes** |
| --- | --- | --- | --- | --- | --- |
| [Development_IGF-RI signaling](http://portal.genego.com/cgi/imagemap.cgi?id=540&filter=1) | Protein function/Growth factors Regulatory processes/Development (common pathways) | [intracellular receptor- mediated signaling pathway, response to extracellular stimulus](http://portal.genego.com/cgi/process.cgi?id=-391082717) | **9.05E-03** | **16** | **44** |
| [Cytoskeleton remodeling_CDC42 in cellular processes](http://portal.genego.com/cgi/imagemap.cgi?id=390&filter=1) | Protein function/G-proteins/ RAS-group Regulatory processes/ Cytoskeleton remodeling | [small GTPase mediated signal transduction](http://portal.genego.com/cgi/process.cgi?id=-1779154733) | **9.31E-03** | **10** | **23** |
| [G-protein signaling_G-Protein alpha-i signaling cascades](http://portal.genego.com/cgi/imagemap.cgi?id=638&filter=1) | Protein function/G-proteins/GPCR | [G-protein coupled receptor protein signaling pathway](http://portal.genego.com/cgi/process.cgi?id=-513419078) | **9.31E-03** | **10** | **23** |
| [G-protein signaling_G-Protein beta/gamma signaling cascades](http://portal.genego.com/cgi/imagemap.cgi?id=641&filter=1) | Protein function/G-proteins/GPCR | [G-protein coupled receptor protein signaling pathway](http://portal.genego.com/cgi/process.cgi?id=-513419078) | **9.31E-03** | **10** | **23** |
| [Development_Mu-type opioid receptor regulation of proliferation](http://portal.genego.com/cgi/imagemap.cgi?id=2424&filter=1) | Protein function/G-proteins/GPCR Regulatory processes/Development/ Neurogenesis | [G-protein coupled receptor protein signaling pathway, response to extracellular stimulus](http://portal.genego.com/cgi/process.cgi?id=-513419078) | **9.31E-03** | **10** | **23** |
| [Development_GDNF signaling](http://portal.genego.com/cgi/imagemap.cgi?id=646&filter=1) | Regulatory processes/Development/ Neurogenesis | [response to extracellular stimulus](http://portal.genego.com/cgi/process.cgi?id=-1078420898) | **1.04E-02** | **9** | **20** |
| [Development_Endothelin-1/EDNRA signaling](http://portal.genego.com/cgi/imagemap.cgi?id=2255&filter=1) | Disease maps/Cardiac Hypertrophy Protein function/G-proteins/GPCR Protein function/Hormones Regulatory processes/Development (common pathways) | [response to hormone stimulus, G-protein coupled receptor protein signaling pathway, response to extracellular stimulus](http://portal.genego.com/cgi/process.cgi?id=-719123304) | **1.07E-02** | **15** | **41** |

**(*Continued*)**

| **Map** | **Map folders** | **Cell process** | ***P*** | **Changed genes** | **Total genes** |
| --- | --- | --- | --- | --- | --- |
| [DNA damage_Role of SUMO in p53 regulation](http://portal.genego.com/cgi/imagemap.cgi?id=648&filter=1) | Regulatory processes/Apoptosis and survival Regulatory processes/ DNA-damage | [apoptosis](http://portal.genego.com/cgi/process.cgi?id=-1971582702) | **1.14E-02** | **8** | **17** |
| [Development_EDG6 signaling pathway](http://portal.genego.com/cgi/imagemap.cgi?id=2952&filter=1) | Protein function/G-proteins/GPCR Regulatory processes/Development (common pathways) | [G-protein coupled receptor protein signaling pathway, response to extracellular stimulus](http://portal.genego.com/cgi/process.cgi?id=-513419078) | **1.20E-02** | **7** | **14** |
| [Proteolysis_Role of Parkin in the Ubiquitin- Proteasomal Pathway](http://portal.genego.com/cgi/imagemap.cgi?id=662&filter=1) | Regulatory processes/Proteolysis | [proteolysis](http://portal.genego.com/cgi/process.cgi?id=-142006563) | **1.31E-02** | **10** | **24** |
| [Apoptosis and survival_Anti-apoptotic action of membrane- bound ESR1](http://portal.genego.com/cgi/imagemap.cgi?id=2736&filter=1) | Protein function/Hormones Regulatory processes/Apoptosis and survival | [response to hormone stimulus, apoptosis](http://portal.genego.com/cgi/process.cgi?id=-719123304) | **1.31E-02** | **10** | **24** |
| [Apoptosis and survival_Anti-apoptotic action of nuclear ESR1 and ESR2](http://portal.genego.com/cgi/imagemap.cgi?id=2737&filter=1) | Protein function/Hormones Regulatory processes/Apoptosis and survival | [response to hormone stimulus, apoptosis](http://portal.genego.com/cgi/process.cgi?id=-719123304) | **1.31E-02** | **10** | **24** |
| [Immune response_MIF - the neuroendocrine- macrophage connector](http://portal.genego.com/cgi/imagemap.cgi?id=518&filter=1) | Regulatory processes/Immune response | [immune response](http://portal.genego.com/cgi/process.cgi?id=-195107408) | **1.33E-02** | **12** | **31** |
| [Development_Endothelin-1/EDNRA transactivation of EGFR](http://portal.genego.com/cgi/imagemap.cgi?id=2254&filter=1) | Protein function/G-proteins/GPCR Protein function/Growth factors Protein function/Hormones Regulatory processes/Development (common pathways) | [response to hormone stimulus, G-protein coupled receptor protein signaling pathway, intracellular receptor-mediated signaling pathway, response to extracellular stimulus](http://portal.genego.com/cgi/process.cgi?id=-719123304) | **1.48E-02** | **13** | **35** |

**(*Continued*)**

| **Map** | **Map folders** | **Cell process** | ***P*** | **Changed genes** | **Total genes** |
| --- | --- | --- | --- | --- | --- |
| [Chemotaxis_Lipoxin inhibitory action on neutrophil migration](http://portal.genego.com/cgi/imagemap.cgi?id=2731&filter=1) | Regulatory processes/Chemotaxis Regulatory processes/Cytoskeleton remodeling Regulatory processes/ Immune response | [immune response](http://portal.genego.com/cgi/process.cgi?id=-195107408) | **1.48E-02** | **13** | **35** |
| [Development _Glucocorticoid receptor signaling](http://portal.genego.com/cgi/imagemap.cgi?id=410&filter=1) | Protein function/Hormones Protein function/Transcription factors Regulatory processes/Development (common pathways) | [response to hormone stimulus, transcription, response to extracellular stimulus](http://portal.genego.com/cgi/process.cgi?id=-719123304) | **1.50E-02** | **9** | **21** |
| [Development_Dopamine D2 receptor transactivation of PDGFR in non-neuronal cells](http://portal.genego.com/cgi/imagemap.cgi?id=2456&filter=1) | Protein function/G-proteins/GPCR Regulatory processes/Development/ Development (common pathways) | [G-protein coupled receptor protein signaling pathway, response to extracellular stimulus](http://portal.genego.com/cgi/process.cgi?id=-513419078) | **1.50E-02** | **9** | **21** |
| [Cytoskeleton remodeling_Fibronectin-binding integrins in cell motility](http://portal.genego.com/cgi/imagemap.cgi?id=451&filter=1) | Regulatory processes/Cell adhesion Regulatory processes/Cytoskeleton remodeling | [cell adhesion](http://portal.genego.com/cgi/process.cgi?id=-552545827) | **1.55E-02** | **11** | **28** |
| [Neurophysiological process_EphB receptors in dendritic spine morphogenesis and synaptogenesis](http://portal.genego.com/cgi/imagemap.cgi?id=528&filter=1) | Regulatory processes/Cell adhesion Regulatory processes/Cytoskeleton remodeling Regulatory processes/ Development/ Neurogenesis Regulatory processes/ Neurophysiological process | [cell adhesion, response to extracellular stimulus](http://portal.genego.com/cgi/process.cgi?id=-552545827) | **1.55E-02** | **11** | **28** |
| [G-protein signaling_G-Protein alpha-s signaling cascades](http://portal.genego.com/cgi/imagemap.cgi?id=640&filter=1) | Protein function/G-proteins/GPCR | [G-protein coupled receptor protein signaling pathway](http://portal.genego.com/cgi/process.cgi?id=-513419078) | **1.55E-02** | **11** | **28** |
| [Transcription_P53 signaling pathway](http://portal.genego.com/cgi/imagemap.cgi?id=412&filter=1) | Protein function/Transcription factors | [transcription](http://portal.genego.com/cgi/process.cgi?id=-929449585) | **1.74E-02** | **12** | **32** |

**(*Continued*)**

| **Map** | **Map folders** | **Cell process** | ***P*** | **Changed genes** | **Total genes** |
| --- | --- | --- | --- | --- | --- |
| [Immune response _IL6 signaling pathway](http://portal.genego.com/cgi/imagemap.cgi?id=479&filter=1) | Protein function/Cyto/chemokines Regulatory processes/Immune response | [cytokine and chemokine mediated signaling pathway, immune response](http://portal.genego.com/cgi/process.cgi?id=-2012554208) | **1.80E-02** | **10** | **25** |
| [Apoptosis and survival_DNA-damage-induced apoptosis](http://portal.genego.com/cgi/imagemap.cgi?id=542&filter=1) | Regulatory processes/Apoptosis and survival | [apoptosis](http://portal.genego.com/cgi/process.cgi?id=-1971582702) | **1.87E-02** | **7** | **15** |
| [Apoptosis and survival_DNA-damage-induced apoptosis](http://portal.genego.com/cgi/imagemap.cgi?id=542&filter=1) | Regulatory processes/Apoptosis and survival | [apoptosis](http://portal.genego.com/cgi/process.cgi?id=-1971582702) | **1.87E-02** | **7** | **15** |
| [Development_Angiotensin signaling via PYK2](http://portal.genego.com/cgi/imagemap.cgi?id=438&filter=1) | Disease maps/Cardiac Hypertrophy Protein function/G-proteins/GPCR Regulatory processes/Development/ Angiogenesis | [response to extracellular stimulus, G-protein coupled receptor protein signaling pathway](http://portal.genego.com/cgi/process.cgi?id=-1078420898) | **1.90E-02** | **13** | **36** |
| [Neurodisease_Parkin disorder under Parkinson's disease](http://portal.genego.com/cgi/imagemap.cgi?id=666&filter=1) | Disease maps/Neurodisease/ Parkinson's Disease |  | **2.05E-02** | **11** | **29** |
| [G-protein signaling_Ras family GTPases in kinase cascades (scheme)](http://portal.genego.com/cgi/imagemap.cgi?id=379&filter=1) | Protein function/G-proteins/RAS- group | [small GTPase mediated signal transduction](http://portal.genego.com/cgi/process.cgi?id=-1779154733) | **2.09E-02** | **9** | **22** |
| [Development_Kappa-type opioid receptor activation of ERK](http://portal.genego.com/cgi/imagemap.cgi?id=2552&filter=1) | Protein function/G-proteins/GPCR Regulatory processes/Development/Development (common pathways) | [G-protein coupled receptor protein signaling pathway, response to extracellular stimulus](http://portal.genego.com/cgi/process.cgi?id=-513419078) | **2.09E-02** | **9** | **22** |
| [Development_Neurotrophin family signaling](http://portal.genego.com/cgi/imagemap.cgi?id=636&filter=1) | Protein function/Growth factors Regulatory processes/Development/ Neurogenesis | [intracellular receptor-mediated signaling pathway, response to extracellular stimulus](http://portal.genego.com/cgi/process.cgi?id=-391082717) | **2.41E-02** | **13** | **37** |

**(*Continued*)**

| **Map** | **Map folders** | **Cell process** | ***P*** | **Changed genes** | **Total genes** |
| --- | --- | --- | --- | --- | --- |
| [Development_A2A receptor signaling](http://portal.genego.com/cgi/imagemap.cgi?id=643&filter=1) | Protein function/G-proteins/GPCR Regulatory processes/Development/Development (common pathways) | [G-protein coupled receptor protein signaling pathway, response to extracellular stimulus](http://portal.genego.com/cgi/process.cgi?id=-513419078) | **2.41E-02** | **13** | **37** |
| [Inhibitory action of Lipoxins on neutrophil migration](http://portal.genego.com/cgi/imagemap.cgi?id=2692&filter=1) | Disease maps/Cystic fibrosis |  | **2.41E-02** | **13** | **37** |
| [DNA damage_NHEJ mechanisms of DSBs repair](http://portal.genego.com/cgi/imagemap.cgi?id=524&filter=1) | Regulatory processes/DNA-damage |  | **2.41E-02** | **8** | **19** |
| [Immune response _Signaling pathway mediated by IL-6 and IL-1](http://portal.genego.com/cgi/imagemap.cgi?id=478&filter=1) | Protein function/Cyto/chemokines Regulatory processes/Immune response | [cytokine and chemokine mediated signaling pathway, immune response](http://portal.genego.com/cgi/process.cgi?id=-2012554208) | **2.42E-02** | **10** | **26** |
| [Cell adhesion_Integrin-mediated cell adhesion](http://portal.genego.com/cgi/imagemap.cgi?id=450&filter=1) | Regulatory processes/Cell adhesion | [cell adhesion](http://portal.genego.com/cgi/process.cgi?id=-552545827) | **2.52E-02** | **14** | **41** |
| [Cytoskeleton remodeling_Reverse signaling by ephrin B](http://portal.genego.com/cgi/imagemap.cgi?id=529&filter=1) | Regulatory processes/Cell adhesion Regulatory processes/Cytoskeleton remodeling Regulatory processes/ Development/Neurogenesis | [cell adhesion, response to extracellular stimulus](http://portal.genego.com/cgi/process.cgi?id=-552545827) | **2.67E-02** | **11** | **30** |
| [Development_Hedgehog and PTH signaling pathways participation in bone and cartilage development](http://portal.genego.com/cgi/imagemap.cgi?id=553&filter=1) | Regulatory processes/Development/ Development (common pathways) | [response to extracellular stimulus](http://portal.genego.com/cgi/process.cgi?id=-1078420898) | **2.67E-02** | **11** | **30** |
| [Development_CNTF receptor signaling](http://portal.genego.com/cgi/imagemap.cgi?id=2231&filter=1) | Protein function/Cyto/chemokines Regulatory processes/Development/ Neurogenesis Regulatory processes/ Hypoxia response | [cytokine and chemokine mediated signaling pathway, response to extracellular stimulus](http://portal.genego.com/cgi/process.cgi?id=-2012554208) | **2.67E-02** | **11** | **30** |

**(*Continued*)**

| **Map** | **Map folders** | **Cell process** | ***P*** | **Changed genes** | **Total genes** |
| --- | --- | --- | --- | --- | --- |
| [Chemotaxis_CXCR4 signaling pathway](http://portal.genego.com/cgi/imagemap.cgi?id=617&filter=1) | Protein function/Cyto/chemokines Protein function/G-proteins/GPCR Regulatory processes/Chemotaxis | [G-protein coupled receptor protein signaling pathway, cytokine and chemokine mediated signaling pathway](http://portal.genego.com/cgi/process.cgi?id=-513419078) | **2.86E-02** | **12** | **34** |
| [G-protein signaling_Proinsulin C-peptide signaling](http://portal.genego.com/cgi/imagemap.cgi?id=2815&filter=1) | Protein function/Hormones | [response to hormone stimulus](http://portal.genego.com/cgi/process.cgi?id=-719123304) | **3.00E-02** | **13** | **38** |
| [Development_A2B receptor: action via G-protein alpha s](http://portal.genego.com/cgi/imagemap.cgi?id=482&filter=1) | Protein function/G-proteins/GPCR Regulatory processes/Development (common pathways) | [G-protein coupled receptor protein signaling pathway, response to extracellular stimulus](http://portal.genego.com/cgi/process.cgi?id=-513419078) | **3.00E-02** | **13** | **38** |
| [DNA damage_Role of NFBD1 in DNA damage response](http://portal.genego.com/cgi/imagemap.cgi?id=650&filter=1) | Regulatory processes/DNA-damage |  | **3.10E-02** | **6** | **13** |
| [Cytoskeleton remodeling_Integrin outside-in signaling](http://portal.genego.com/cgi/imagemap.cgi?id=664&filter=1) | Regulatory processes/Cell adhesion Regulatory processes/Cytoskeleton remodeling | [cell adhesion](http://portal.genego.com/cgi/process.cgi?id=-552545827) | **3.18E-02** | **15** | **46** |
| [Immune response _Oncostatin M signaling via JAK-Stat in human cells](http://portal.genego.com/cgi/imagemap.cgi?id=2207&filter=1) | Protein function/Cyto/chemokines Regulatory processes/Immune response/Immune response: organism-specific maps for Mouse, Rat and Human | [cytokine and chemokine mediated signaling pathway, immune response](http://portal.genego.com/cgi/process.cgi?id=-2012554208) | **3.33E-02** | **8** | **20** |
| [Cytoskeleton remodeling_Role PKA in cytoskeleton reorganisation](http://portal.genego.com/cgi/imagemap.cgi?id=543&filter=1) | Protein function/Kinases Regulatory processes/Cytoskeleton remodeling | [protein kinase cascade](http://portal.genego.com/cgi/process.cgi?id=-2009044596) | **3.41E-02** | **11** | **31** |

**(*Continued*)**

| **Map** | **Map folders** | **Cell process** | ***P*** | **Changed genes** | **Total genes** |
| --- | --- | --- | --- | --- | --- |
| [Development_EPO-induced MAPK pathway](http://portal.genego.com/cgi/imagemap.cgi?id=738&filter=1) | Protein function/Growth factors Regulatory processes/Development/ Hemopoiesis | [intracellular receptor- mediated signaling pathway, response to extracellular stimulus](http://portal.genego.com/cgi/process.cgi?id=-391082717) | **3.70E-02** | **13** | **39** |
| [Regulation of lipid metabolism_Alpha-1 adrenergic receptors signaling via arachidonic acid](http://portal.genego.com/cgi/imagemap.cgi?id=2385&filter=1) | Protein function/G-proteins/GPCR Regulation of metabolism/Regulation of lipid metabolism | [G-protein coupled receptor protein signaling pathway](http://portal.genego.com/cgi/process.cgi?id=-513419078) | **3.70E-02** | **13** | **39** |
| [Signal transduction_PKA signaling](http://portal.genego.com/cgi/imagemap.cgi?id=675&filter=1) | Protein function/G-proteins/GPCR Protein function/Kinases Protein function/Second messenger | [G-protein coupled receptor protein signaling pathway, second-messenger-mediated signaling, protein kinase cascade](http://portal.genego.com/cgi/process.cgi?id=-513419078) | **3.76E-02** | **9** | **24** |
| [Immune response _CD16 signaling in NK cells](http://portal.genego.com/cgi/imagemap.cgi?id=2249&filter=1) | Regulatory processes/Immune response | [immune response](http://portal.genego.com/cgi/process.cgi?id=-195107408) | **4.52E-02** | **17** | **56** |
| [Development_Prolactin receptor signaling](http://portal.genego.com/cgi/imagemap.cgi?id=545&filter=1) | Protein function/Growth factors Protein function/Hormones Regulatory processes/Development/Development (common pathways) | [response to hormone stimulus, intracellular receptor-mediated signaling pathway, response to extracellular stimulus](http://portal.genego.com/cgi/process.cgi?id=-719123304) | **4.55E-02** | **16** | **52** |
| [Proteolysis_Putative SUMO-1 pathway](http://portal.genego.com/cgi/imagemap.cgi?id=699&filter=1) | Regulatory processes/Proteolysis | [proteolysis](http://portal.genego.com/cgi/process.cgi?id=-142006563) | **4.85E-02** | **9** | **25** |

2.00-fold change of gene expression by PAX3 siRNA#4 transfection compared with non-targeting control siRNA was used as a threshold.

**Supplementary Table S6** Alterations of gene expression (fold change) in PAX3 down-regulated neuroblastoma cells compared with microarray data from PAX3 over-expressed cells

| **Gene symbol** | **PAX3 down-regulated cells** | | | | **PAX3 over-expressed**  **myoblasts** | | | **PAX3 over-expressed**  **melanocytes** | | | **PAX3 over-expressed**  **stem cells** | | |
| --- | --- | --- | --- | --- | --- | --- | --- | --- | --- | --- | --- | --- | --- |
|  | **SH-EP1** | | **SH-SY5Y** | |  |  |  |  |  |  |  |  |  |
|  | **Microarray q-PCR** | | **Microarray q-PCR** | | **PAX3c** | **PAX3e** | **PAX3g** | **PAX3c** | **PAX3e** | **PAX3g** | **PAX3c** | **PAX3e** | **PAX3g** |
| **IL6ST** | **6.88** | **5.62** | **2.49** | **3.36** | **-1.65** | **1.07** | **-2.05** |  |  |  |  |  |  |
| **MDM2** | **5.27** | **-1.09** | **2.33** | **8.99** | **-2.04** | **-2.91** | **-1.49** |  |  |  |  |  |  |
| **IGFBP3** | **4.32** | **2.76** | **1.58** | **1.54** | **-127.38** | **-20.10** | **-59.36** |  |  |  |  |  |  |
| **SMAD2** | **2.48** | **3.01** | **1.81** | **2.07** | **-1.86** | **-2.49** | **-1.24** |  |  |  |  |  |  |
| **NID1** | **1.90** | **1.79** | **2.03** | **2.74** | **-9.90** | **-10.58** | **-10.96** |  |  |  |  |  |  |
| **MCM7** | **-65.26** | **-7.20** | **-1.74** | **-1.28** | **3.21** | **3.07** | **5.82** |  |  |  |  |  |  |
| **GTSE1** | **-54.15** | **-23.32** | **-2.72** | **-1.41** | **8.40** | **3.28** | **21.41** |  |  |  |  |  |  |
| **AURKB** | **-45.82** | **-14.16** | **-2.56** | **-1.45** | **22.04** | **29.00** | **67.78** |  |  |  |  |  |  |
| **BIRC5** | **-39.38** | **-12.18** | **-2.77** | **-1.42** | **39.19** | **24.84** | **71.46** |  |  |  |  |  |  |
| **BUB1** | **-38.15** | **-9.09** | **-1.46** | **-1.03** | **51.59** | **61.81** | **137.01** |  |  |  |  |  |  |
| **AURKA** | **-32.95** | **-7.64** | **-2.97** | **-1.31** | **3.33** | **3.80** | **11.91** |  |  |  |  |  |  |
| **CDCA3** | **-31.74** | **-12.57** | **-1.76** | **-1.11** | **6.26** | **4.50** | **11.65** |  |  |  |  |  |  |
| **HMMR** | **-22.39** | **-9.38** | **-1.67** | **-1.35** | **5.02** | **4.37** | **11.57** |  |  |  |  |  |  |
| **CCNA2** | **-19.41** | **-11.99** | **-1.60** | **-1.28** | **14.06** | **15.35** | **28.66** | **1.56** | **2.78** | **1.95** |  |  |  |
| **BRCA1** | **-9.88** | **-6.00** | **-1.34** | **-1.27** | **23.30** | **18.26** | **56.66** |  |  |  |  |  |  |
| **POLA2** | **-8.49** | **-4.15** | **-2.01** | **1.08** | **4.41** | **3.74** | **7.10** |  |  |  |  |  |  |
| **PLK1** | **-7.45** | **-12.87** | **-2.80** | **-1.07** | **5.43** | **8.88** | **22.56** |  |  |  |  |  | **10.69** |
| **CDT1** | **-5.44** | **-6.85** | **-2.63** | **1.15** | **ND** | **ND** | **ND** |  |  |  | **2.00** |  |  |
| **MITF** | **-1.99** | **-2.15** | ND | **-1.20** | **ND** | **ND** | **ND** | **6.67** | **1.10** | **1.56** |  |  |  |
| **BCL2** | **1.14** | **-1.60** | **2.37** | **3.03** | **11.81** | **5.02** | **7.57** |  |  |  |  |  |  |

ND, not detected. 1.50-fold change of gene expression was used as a threshold. Gene expression up-regulated > 1.50-fold is shown in red; gene expression down-regulated > 1.50-fold is shown in blue; in black means no change.
